# Circ-TRIO promotes TNBC progression by regulating the miR-432-5p/CCDC58 axis

**DOI:** 10.1038/s41419-022-05216-7

**Published:** 2022-09-08

**Authors:** Zekun Wang, Yaming Li, Jingwen Yang, Yiran Liang, Xiaolong Wang, Ning Zhang, Xiaoli Kong, Bing Chen, Lijuan Wang, Wenjing Zhao, Qifeng Yang

**Affiliations:** 1 Department of Breast Surgery, General Surgery, Qilu Hospital of Shandong University, Jinan, China; 2 Pathology Tissue Bank, Qilu Hospital of Shandong University, Jinan, China; 3 Research Institute of Breast Cancer, Shandong University, Jinan, China

**Keywords:** Breast cancer, Cancer

## Abstract

Numerous studies have shown that circRNAs are aberrantly expressed in various cancers and play a significant role in tumor progression. However, the molecular mechanisms of circRNAs in triple-negative breast cancer (TNBC) remain ambiguous. By intersecting throughput data and qRT-PCR results from tissues and cell lines, circ-TRIO was identified as a potential oncogenic regulator of TNBC. Moreover, circ-TRIO expression was detected in TNBC tissues and was correlated with the recurrence and prognosis of TNBC patients. The circular characteristics of circ-TRIO were verified by RNase R and CHX assays. Functionally, the knockdown of circ-TRIO inhibited the proliferation, migration and invasion of TNBC cells, while the overexpression of circ-TRIO resulted in the opposite impacts. Mechanistically, a dual luciferase reporter assay and RNA immunoprecipitation were performed and indicated that circ-TRIO could combine with miR-432-5p to regulate the expression of coiled-coil domain containing 58 (CCDC58). In summary, our study illustrates that circ-TRIO plays an important role in the progression of TNBC by regulating the miR-432-5p/CCDC58 axis, which could broaden our insight into the underlying mechanisms and provide a novel prognostic marker of TNBC in the clinic.

## Introduction

In recent years, breast cancer has become the most common malignancy worldwide and seriously affects women’s physical and mental health [1, 2]. According to the latest report by the International Agency for Research on Cancer (IARC), breast cancer first exceeded the incidence of lung cancer and accounted for 11.7% of new cancer cases in 2020 [3]. Breast cancer is a heterogeneous disease based on the expression of the estrogen receptor (ER), progesterone receptor (PR) and human epidermal growth factor receptor-2 (HER2), which can be divided into five entirely different subtypes, including luminal A, luminal B, Her-2 overexpressed, normal breast-like and triple-negative breast cancer (TNBC) [4, 5]. As reported in previous studies, TNBC is regarded as the most aggressive subtype due to its high speed of proliferation, metastasis and lack of endocrine targets, resulting in a poorer prognosis compared with hormone-positive BC [6, 7]. Since rapid proliferation and early metastasis are essential factors that contribute to the poor prognosis of TNBC, it is extremely urgent to determine the molecular mechanisms and identify new targets for TNBC progression.

In recent years, an increasing number of noncoding RNAs have been discovered in mammalian tissues and cells, such as lncRNAs, miRNAs and circRNAs [8]. CircRNAs constitute a special class of endogenous noncoding RNAs that differ from linear RNAs containing a 5′ m [7] G cap and 3′ poly(A) tail, and circRNAs possess covalently closed single-stranded circular structures by back-splicing precursor mRNAs [9]. Due to their special circular structure, circRNAs are resistant to exonuclease-mediated degradation and have a longer half-life [10]. Moreover, it has been reported that circRNAs are abundantly expressed in eukaryotes and have evolutionary sequence conservation and developmental stage-specific features, indicating that circRNAs might play vital roles in the biological functions of cells [11–13]. Studies have also shown that circRNAs are related to human diseases, such as cancers [14] and have become a hot topic in cancer research [5, 15]. CircRNAs can exert their functions in cancers via different approaches, such as by regulating the activity of downstream mRNAs by sponging miRNAs [16], which have been reported to play vital roles in the malignant behaviors of cancers, including tumorigenesis, proliferation, metastasis, and apoptosis [14, 16]. In breast cancer, circRNAs, such as circPGR6 [17], circKDM4B [18], and circTADA2As [5], are also associated with tumorigenesis and progression in combination with miRNAs. However, the functions and mechanisms of circRNAs in TNBC remain uncertain and need further exploration.

In the present study, we identified a novel dysregulated circRNA termed circ-TRIO (genomic location: chr5: 14316621-14336836; circBase [19] ID: hsa_circ_0005260) in TNBC [20]. We found that circ-TRIO is highly expressed in cancer tissues and metastatic cell lines and is correlated with the recurrence and prognoses of TNBC patients. The functions and mechanisms of the circ-TRIO/miR-432-5p/CCDC58 axis on TNBC cell proliferation and metastasis were further evaluated in vitro and in vivo. In conclusion, we attempted to perform a systematic and comprehensive functional analysis and explore its regulatory capacity on the expression of target genes to fill in the gaps in research investigating the underlying function of circ-TRIO in TNBC progression.

## Materials and methods

### Ethics statement and human tissue samples

All of our procedures of experiments have been given permission by the Ethical Committee of Shandong University. TNBC tissues used in this study were collected from the TNBC patients admitted to Qilu Hospital, which were confirmed by pathology and further stored at −80 °C after surgery. The written, informed consent for the use of clinical information and samples in this research were obtained from all patients.

### CircRNA sequencing

TNBC cell line MDA-MB-231 and its metastatic subcell line 231_M were used for circRNA sequencing analysis. The preparation and sequencing of samples were performed by Cloud-Seq Biotech (Shanghai, China). CircRNAs with fold change ≥2 and *P* value < 0.05 were considered as significant differentially expressed.

### Cell culture and reagent

All cell lines used in this study except 231_M were obtained from the American Type Culture Collection (ATCC) and were cultured according to the manufacturer’s instructions. Briefly, MCF10A, MCF10AT, MCF10CA1A and MCF10CA1H cell lines were cultured in Dulbecco’s modified Eagle’s medium (Invitrogen, USA) with 5% horse serum, 10 μg/ml insulin, 20 ng/ml EGF, 100 ng/ml cholera toxin, and 0.5 μg/ml hydrocortisone. SK-BR-3, MCF7, MDA-MB-468, HS578T, MDA-MB-231, 231_M and HEK293T cell lines were cultured in DMEM medium (DMEM; Invitrogen, Carlsbad, CA, USA) containing 10% fetal bovine serum (FBS; HyClone). T47D cells were cultured with RPMI 1640 medium. All cell lines were cultured in a 5% CO_2_ humidified incubator at 37 °C.

### Generation of the MDA-MB-231 subcell line with high metastatic potential (231_M)

The generation procedures and metastatic properties of 231_M have been reported in our previously published article [21]. In brief, MDA-MB-231 cells were injected into the lateral tail veins of 4– to 5-week-old BALB/c nu/nu female mice. Eight weeks later, the mice were sacrificed, and the lungs were extracted. Then, MDA-MB-231 cells that had metastasized to the lung were filtered by flow cytometry and further cultured and expanded in vitro. The same experimental procedures were used to generate a second-round subcell line, which was designated 231_M in this article.

### RNA extraction, nuclear-cytoplasmic fractionation and RT-PCR

Total RNA was extracted from TNBC tissues or cells by TRIzol Reagent (Invitrogen, Carlsbad, CA USA), and RNAs from nucleus and cytoplasm of TNBC cells were separated by the PARIS™ Kit (Invitrogen, Carlsbad, CA, USA). The PrimeScript RT Reagent Kit (Takara, Shiga, Japan) was then used to synthesize cDNA from extracted RNA. For miRNAs, reverse transcription was performed using the PrimeScript miRNA cDNA Synthesis Kit (TaKaRa, Japan). Quantitative real-time PCR (qRT-PCR) was conduct by using SYBR Green PCR mix (Takara, Shiga, Japan). The primers used in this study were listed as supplementary Table S.2. β-actin was used as an endogenous control for circRNAs and mRNA, and U6 was used as an endogenous control for miRNA. The relative expression levels of genes were analyzed by the standard 2^-ΔΔCt^ method.

### Actinomycin D and RNase R treatment

When performing Actinomycin D treatment assay, 2 μg/mL Actinomycin D (Sigma) was added to the culture medium of MDA-MB-231 and MDA-MB-468, then the total RNA was extracted from TNBC cells after 0, 3, 6, 12 and 24 h of treatment. For RNase R treatment assay, total RNA from TNBC cells were divided into two parts and incubated with or without 3 U/mg RNase R for 15 min at 37 °C. After treatment with actinomycin D and RNase R, the RNA expression levels of circ-TRIO and TRIO were further evaluated using qRT-PCR.

### Cell proliferation assay

Breast cancer cells were seeded into the 96-well plate at a concentration of 2 × 10^3^ cells per well. At the indicated time points, the cells were incubated with 20 μL of sterile MTT for 4 h at 37 C, after which the medium was removed and replaced with 100 μL of DMSO. Then we detected the absorbance value of cells at 450 nm using microplate reader.

### EDU assay

EDU Proliferation Kit (RiboBio Guangzhou, China) was used in EDU assay. Firstly, 1 × 10^4^ cells were seeded into 96-well plate after transfection. After cell attachment, 50 mM EDU was added to cells and incubated for 2.5 h, then the cells were fixed with 4% paraformaldehyde (PFA) and further stained with Apollo Dye Solution. After that, Hoechst was selected to label nucleic acid. Images were obtained with an Olympus microscope (Olympus, Tokyo, Japan).

### Colony formation assay

The breast cancer cells of experimental group and control group were seeded into six-well plates at a concentration of 800 cells per well. We then placed the cells in the incubator at 37 °C for approximately 10 days until dot clones were visible to the naked eye. After that, cells were washed with PBS and fixed with methanol, then further stained with crystal violet solution.

### Cell cycle assays

Transfected cells were seeded into the six-well plate at a concentration of 2 × 10^5^ cells per well. After 48 h of culture, cells were digested with Trypsin (Beyotime, Shanghai, China) and washed with PBS for twice. After that, cells were fixed with ice-cold 75% ethanol in PBS and stained with 500 μl DNA staining solution for 30 min (Multisciences, Shanghai, China). The cells were analyzed via flow cytometry.

### Migration and invasion assays

Both migration and invasion assays were performed by using the Transwell system (Corning Costar, Lowell, MA, USA). First of all, cells were digested and washed by PBS for three times, then resuspended by culture medium without serum. In the migration system, 700 μL of culture medium with 20% FBS was added to the lower well of each chamber, and 1 × 10^5^ cells suspended in serum-free medium were added to the upper inserts. For invasion assay, Matrigel (BD Biosciences, Bedford, MA, USA) needed to be added to the membrane of upper inserts before adding cells. After specified time for incubation, the total number of cells which have traversed the membrane would be quantified.

### Wound-healing assay

The number of transfected cells which was seeded in 24-well plates should be 3 × 10^5^ per-well, then they would be cultured in DMEM with 10% FBS for around 24 h until a confluent monolayer had formed. Then a sterile 10 μl plastic pipette tip was selected to make scratches on the monolayer, followed by washed with PBS to remove the detached cells. Cells were cultured in DMEM without FBS to eliminate the influence of proliferation, and images were captured at the indicated times (0 and 48 h) using an Olympus light microscope.

### Fluorescence in situ hybridization (FISH)

FISH assay was conducted by using FISH kit (GenePharma, Shanghai, China) to indicate the subcellular location of circ-TRIO in TNBC cells. After prehybridization at 73 °C for 5 min, then cells were hybridized with specific Cy3-labeled circ-TRIO probes at 37 °C overnight, and cell nuclei were stained with Hoechst. Fluorescence microscope (Leica, Wetzlar, Germany) was used to take photographs.

### Luciferase reporter assay

To evaluate the direct combination between circ-TRIO and miR-432-5p, full sequence of circ-TRIO and its mutant version were constructed into pMIR-GLO vector. HEK-293T cells were co-transfected with miR-432-5p mimics and wild type or mutant type of pMIR-GLO vectors. After 48 h of incubation, luciferase reporter assays were performed by using a Dual Luciferase Assay System Kit (Promega, Madison, WI, USA) according to the manufacturer’s instructions. The wild type and mutant type of CCDC58 3ʹ UTR were also cloned into the pMIR-GLO vector to validate combination between miR-432-5p and CCDC58. The subtracted difference of firefly and Renilla luciferase activities was calculated as relative luciferase activity.

### Protein isolation and western blot

Total proteins were extracted, separated by 10% SDS-PAGE gel, and then transferred to 0.22 μm polyvinylidine difluoride (PVDF) membranes (Millipore, Billerica, MA, USA). The PVDF membranes were incubated with 5% skim milk to block nonspecific binding at room temperature for 1 h. Then the membranes were incubated with primary antibodies overnight at 4 °C and secondary antibodies for 2 h at room temperature. The antibodies used in this study are presented in Table S.3. The protein expression levels were detected by chemiluminescence (Millipore, Billerica, MA, USA). β-actin was used as endogenous control.

### RIP assay

RIP assays were performed by using the Magna RIP RNA-Binding Protein Immunoprecipitation Kit (Millipore, Billerica, MA, USA), following to the instructions of manufacturer. Antibodies were purchased from Millipore, which were applied for RIP assays against Ago2 and immunoglobulin G (IgG). Furthermore, total RNA was extracted for the detection of circRNA and miRNA. Finally, the expressions of circ-TRIO and miR-432-5p were examined by qRT-PCR.

### Tumor xenograft model

MDA-MB-231 cells that stably expressed pLCDH or circ-TRIO (1 × 10^7^ cells) were suspended in 200 μL of PBS and injected subcutaneously into the left flanks of 4- to 6-week-old BALB/c nu/nu female mice (randomly divided 5 mice in each group). The growth rates of the tumors were monitored by measuring the maximum (L) and minimum (W) length every 7 days after the initial week. All mice were killed after 4 weeks, the tumors were collected, and the weights were measured. The tumor volume was calculated as ½LW^2^.

To evaluate the effects of circ-TRIO on lung metastasis, 1 × 10^5^ cells were injected into the tail veins of mice (randomly divided 5 mice in each group). After 4 weeks, all mice were killed under anesthesia, and the lungs were collected to evaluate the number of pulmonary metastatic lesions. H&E staining was performed on sections from embedded samples for the tissue morphology evaluation. Lung metastasis was also monitored by a Xenogen IVIS Spectrum Imaging System (PerkinElmer, USA) after intraperitoneal injection.

### Immunohistochemistry (IHC)

Tissues separated from nude mice were fixed with formalin for at least 24 h. After that the tissues were paraffin-embedded and then sliced into 4-µm section for further IHC. Primary antibodies including N-cad (1:200), Ki-67(1:200) and CCDC58(1:200) were used to incubate paraffin slices at 4°C overnight, following by peroxidase-conjugated secondary antibody for 2 h at room temperature. Then the tissue sections were stained with diaminobenzidine, and counterstained with hematoxylin. Olympus light microscope was used to take photos for the representative areas.

### Statistical analysis

Our results were represented at least three independently performed experiments. The SPSS software (version 18.0) was used for the statistical analysis. Two group comparisons were performed with the student’s *t* test. All the performed tests were two-sided and error bars represent the standard error of the mean (SEM) of experiments. Differences with *p* < 0.05 were considered to be statistically significant.

## Results

### Identification and characteristics of circ-TRIO in TNBC

**Fig. 1:**
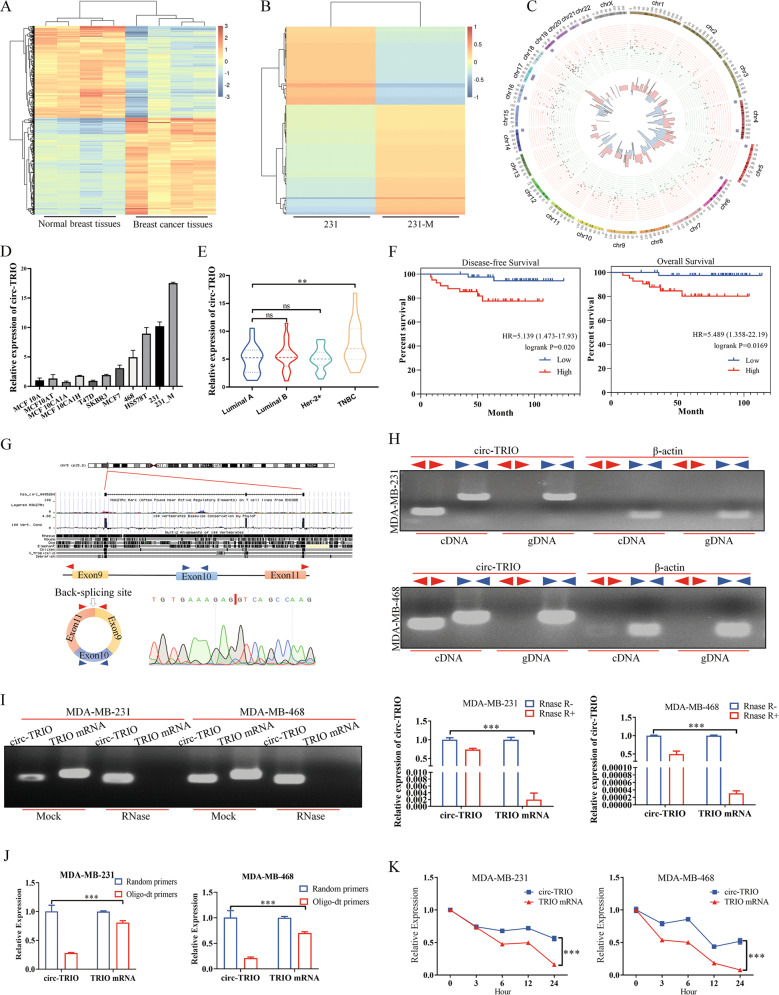
Identification and characterization of circ-TRIO as a novel potential oncogene in TNBC. **A** Heatmap of the significantly differentially expressed circRNAs between breast cancer tissues and normal breast tissues (red indicates upregulation of circRNAs, and blue indicates downregulation of circRNAs). **B** Heatmap of the significantly differentially expressed circRNAs between the 231 and 231_M cell lines (red indicates upregulation of circRNAs, and blue indicates downregulation of circRNAs). **C** Circos diagrams showing the characteristic data and their relationships at various levels across the entire genome. **D** The relative expression of circ-TRIO was measured in different breast cancer cell lines using quantitative real-time PCR. **E** Expression levels of circ-TRIO in different subtypes of breast cancer (*n* = 20 per group). **F** Tissues from a total of 84 TNBC patients were collected, and a Kaplan‒Meier analysis was performed to evaluate the association between circ-TRIO expression and the prognosis of TNBC patients. **G** Upper panel: A schematic diagram indicating the genomic loci of circ-TRIO. Lower panel: Divergent and convergent primers of circ-TRIO used in this study, and Sanger sequencing following PCR conducted using the indicated divergent flanking primers confirmed the “head-to-tail” splicing of circ-TRIO. **H** RT‒PCR assay with divergent or convergent primers indicated that circ-TRIO existed in cDNA but not in gDNA. β-actin was used as a negative control. **I** Total RNA from TNBC cells treated with or without RNase-R was subjected to polymerase chain reaction (PCR). The relative expression of circ-TRIO and TRIO mRNA was detected by real-time PCR. **J** The relative expression levels of circ-TRIO and TRIO mRNA in TNBC cells were analyzed by qRT‒PCR with random primers and oligo (dT) primers. **K** qRT‒PCR indicated the abundance of circ-TRIO and TRIO mRNA after treatment with actinomycin D at the indicated time points in TNBC cells. ns nonsignificant; **P < 0.01; ***P < 0.001.

To screen potential functional circRNAs associated with the progression of TNBC, circRNA array data from the GEO database (GEO: GSE165884) were first used to filter the differentially expressed circRNAs between breast cancer tissues and normal breast tissues. As shown in Fig. 1A, in total, 454 circRNAs were significantly changed, with 233 circRNAs upregulated and 221 circRNAs downregulated in breast cancer tissues. Furthermore, MDA-MB-231 and its subcellular line 231_M (MDA-MB-231 with higher metastatic potential) were selected for circRNA-seq to screen candidate circRNAs (Fig. 1B). We found that 241 circRNAs were remarkably altered in the 231_M subcell line. Further intersecting the significantly changed circRNAs based on the above two results, only 5 circRNAs were upregulated in both breast cancer tissues and 231_M, indicating that these 5 circRNAs might play a role in breast cancer progression (Fig. 1C). We present the basic information of the filtered circRNAs in Table S.1. Subsequently, qRT‒PCR was used to evaluate the expression levels of the filtered circRNAs, and we found that circ-TRIO was significantly upregulated in the TNBC cell lines and positively correlated with the degree of malignancy of breast cancer cells (Fig. 1D); additionally, circ-TRIO was specifically upregulated in the TNBC tissues compared with tissues of the other subtypes (luminal A, luminal B and HER2 enriched) (Fig. 1E), suggesting that circ-TRIO might be associated with the malignant behaviors of TNBC. Furthermore, 84 TNBC patients were randomly selected and equally divided into two groups based on the expression of circ-TRIO. The association between the expression of circ-TRIO and the basic characteristics of the TNBC patients is shown in Table 1, and circ-TRIO expression was correlated with recurrence in the patients. Moreover, we found that circ-TRIO expression was significantly correlated with both disease-free survival (DFS) and overall survival (OS) in TNBC patients, and higher circ-TRIO expression predicted a poorer prognosis (Fig. 1F). Univariate and multivariate analyses of both DFS and OS were also performed, and these analyses further indicated that the expression of circ-TRIO could be an independent prognostic predictor of both DFS (Table 2) and OS (Table 3). Based on the aforementioned results, circ-TRIO was finally selected as the potential functional circRNA in our study.

**Table 1:** Association between clinicopathological variables and circ-TRIO expression in TNBC patients.

Variable	Circ-TRIO expression		*P* value
	Low	High	
Age			0.053
Age ≤45	16	8	
Age >45	26	34	
Histological grade			0.494
G2	22	21	
G3	20	20	
Unknown	0	1	
Tumor size			0.281
≤2 cm	12	17	
>2 cm	29	25	
Unknown	1	0	
Lymph node status			0.369
Negative	28	24	
Positive	14	18	
KI67 status			0.693
Low	3	4	
High	39	38	
Recurrence			**0.024**
No	40	33	
Yes	2	9	

Bold values indicates statistical significant *P* values.

**Table 2:** Univariate and multivariate analyses of prognostic factors (DFS) for patients with TNBC.

Variable	Univariate analysis (DFS)		Multivariate analysis (DFS)	
	HR (95% CI)	*P* value	HR (95% CI)	*P* value
Age				
Age≤45	Reference	–		
Age>45	1.741 (0.369–8.213)	0.483		
Histological grade				
G2	Reference	–		
G3	0.729 (0.206–2.584)	0.625		
Unknown	–	–		
Tumor size				
≤2 cm	Reference	–		
>2 cm	2.418 (0.513–11.395)	0.264		
Unknown	–	–		
Lymph node status				
Negative	Reference	–	Reference	–
Positive	**4.351 (1.123–16.850)**	**0.033**	**4.090 (1.053–15.888)**	**0.042**
KI67 status				
Low	Reference	–		
High	0.424			
circ-TRIO expression				
Low	Reference	–	Reference	–
High	**5.248 (1.106–24.902)**	**0.037**	**4.961 (1.040–23.665)**	**0.044**

Bold values indicates statistical significant *P* values.

**Table 3:** Univariate analyses of prognostic factors (OS) for patients with TNBC.

Variable	Univariate analysis (OS)	
	HR (95% CI)	*P* value
Age		
Age≤45	Reference	–
Age>45	1.326 (0.267–6.576)	0.73
Histological grade		
G2	Reference	–
G3	3.355 (0.677–16.631)	0.138
Unknown	–	–
Tumor size		
≤2 cm	Reference	–
>2 cm	43.901 (0.105–18354.335)	0.219
Unknown	–	–
Lymph node status		
Negative	Reference	–
Positive	4.975 (1.003–24.671)	0.051
KI67 status		
Low	Reference	–
High	23.550 (0.001–71774.760)	0.549
circ-TRIO expression		
Low	Reference	–
High	**8.477 (1.038–69.205)**	**0.046**

Bold values indicates statistical significant *P* values.

On the basis of the circBase database, the genomic location of circ-TRIO is chr5:14,316,621–14,336,836 (Fig. 1G, upper panel); circ-TRIO is generated by the back-splicing of 9–11 exons of the triple functional domain (TRIO) gene, and ultimately, the spliced mature sequence has a length of 546 nt (Fig. 1G, lower left panel). Specific convergent (blue) and divergent (red) primers for circ-TRIO were designed, and the specific joint sequence of head-to-tail splicing was detected in TNBC cells (Fig. 1G, lower right panel). cDNA and gDNA obtained from MDA-MB-231 and MDA-MB-468 cells were subjected to PCR and electrophoresis, and the results showed that circ-TRIO was only amplified by divergent primers in the cDNA but not the extracted gDNA (Fig. 1H). Moreover, Fig. 1I indicates that circ-TRIO was resistant to the digestion of ribonuclease R (RNase R); as shown in Fig. 1J, circ-TRIO did not contain a poly(A) tail, confirming the circular isoform of circ-TRIO. In addition, MDA-MB-231 and MDA-MB-468 cells were treated with actinomycin D, an inhibitor of RNA synthesis, which revealed that the half-life of circ-TRIO was much longer than that of the linear form TRIO mRNA (Fig. 1K). In conclusion, our results demonstrate that circ-TRIO is an endogenously expressed circular RNA in TNBC cells.

### Knockdown of circ-TRIO inhibits the proliferation, migration, and invasion of TNBC cells

**Fig. 2:**
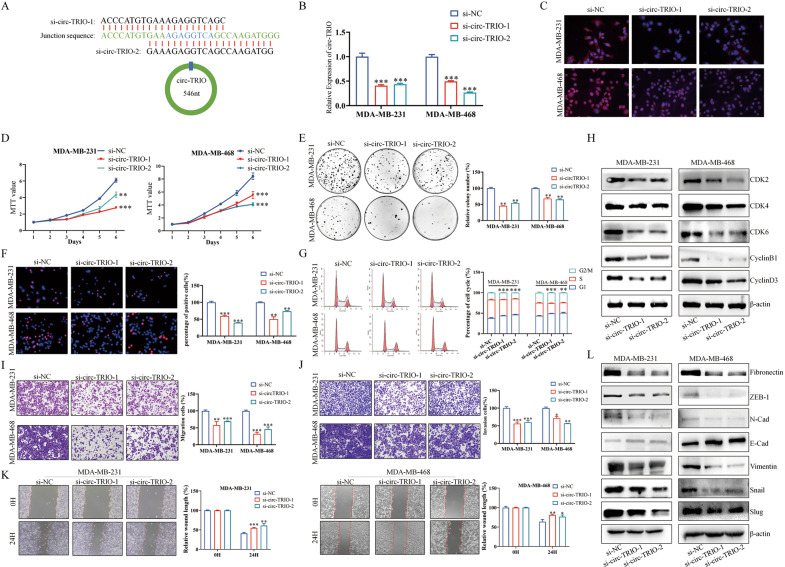
Knockdown of circ-TRIO suppresses the proliferation, migration, and invasion of TNBC cells. **A** Schematic illustration showing the sequences of the siRNAs targeting the back-splicing junction sequence of circ-TRIO. **B** qRT‒PCR was used to examine the inhibition efficiency of the siRNAs. **C** Fish assay revealed the inhibition efficiency of the siRNAs. MTT (**D**) and colony formation assays (**E**) were used to assess the proliferation rate of TNBC cells after the knockdown of circ-TRIO. EdU (**F**) and flow cytometry (**G**) assays were performed to evaluate the effects of circ-TRIO inhibition on the proliferation activity of breast cancer cells. **H** The expression of representative cell cycle-related proteins was revealed by Western blot assays. The migration (**I**) and invasion (**J**) abilities of the MDA-MB-231 and MDA-MB-468 cell lines after the knockdown of circ-TRIO were evaluated by using Transwell assays. **K** Wound-healing assays were performed to identify the migration ability. **L** Western blot assays were performed to evaluate the expression changes of EMT pathway-related proteins after circ-TRIO knockdown. *p < 0.05; **p < 0.01; ***p < 0.001.

To investigate the potential roles of circ-TRIO in TNBC, two siRNAs (si-circ-TRIO-1 and si-circ-TRIO-2) specifically targeting the back-splicing junction of circ-TRIO were designed to knockdown the expression of circ-TRIO, and the position and sequences of both siRNAs are shown in Fig. 2A. QRT-PCR (Fig. 2B) and FISH (Fig. 2C) assays were performed to evaluate the knockdown specificity and efficiency of the siRNAs. In addition, the expression of TRIO mRNA was examined, and no significant changes were observed after the circ-TRIO knockdown, indicating that the subsequent experiments did not result from the nonspecific knockdown of TRIO mRNA (Fig. S1A). MTT (Fig. 2D) and colony formation (Fig. 2E) assays were performed, and we found that the downregulation of circ-TRIO could significantly inhibit the proliferation of TNBC cells. EdU and cell cycle assays were further conducted, and we found that the proliferation activities of TNBC cells were suppressed after circ-TRIO deficiency by triggering G1 phase arrest of the cell cycle (Fig. 2F, G). Western blot assays further demonstrated that the knockdown of circ-TRIO decreased the protein levels of cell cycle-related proteins (Fig. 2H). The above results imply that circ-TRIO deficiency could suppress the proliferation of TNBC cells by triggering G1 phase arrest of the cell cycle.

Subsequently, we also examined the effects of circ-TRIO knockdown on TNBC cell migration and invasion. Transwell assays and wound healing assays showed that the migration and invasion abilities of TNBC cells were significantly suppressed after the knockdown of circ-TRIO (Fig. 2I–K). Because the epithelial-mesenchymal transition is essential for the metastatic abilities of tumor cells [22], the protein levels of epithelial and mesenchymal (EMT) markers were further examined, and we found that the knockdown of circ-TRIO contributed to the inhibition of the EMT process (Fig. 2L). Taken together, our results prove that the knockdown of circ-TRIO inhibited the migration and invasion abilities of TNBC cells by suppressing the EMT process.

### Overexpression of circ-TRIO enhances the malignant abilities of TNBC

**Fig. 3:**
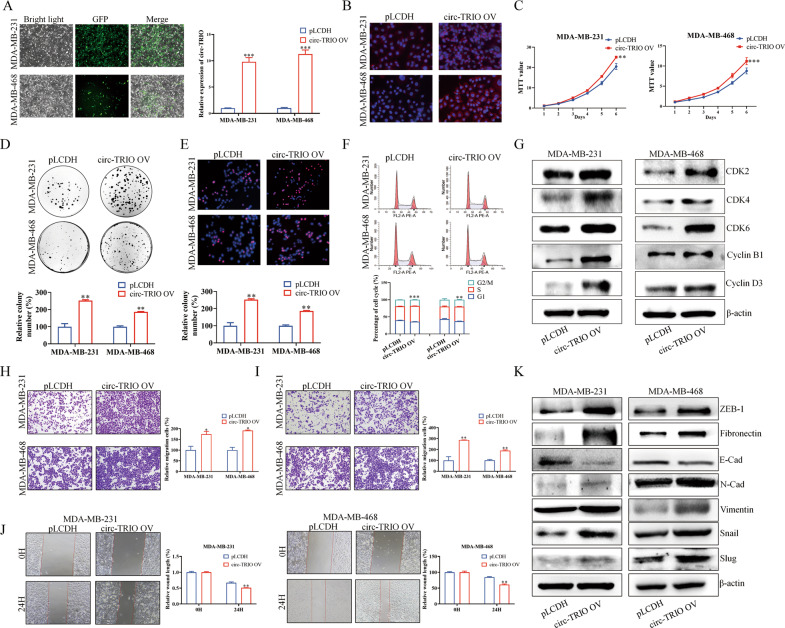
Overexpression of circ-TRIO promotes proliferation, migration, and invasion of TNBC cells. **A** The efficiency of circ-TRIO overexpression was verified by microscopy and qRT‒PCR. **B** FISH assay was performed to examine the efficiency of circ-TRIO overexpression. MTT (**C**) and flat plate colony formation assays (**D**) were used to assess the proliferation rate of TNBC cells after the upregulation of circ-TRIO. EdU (**E**) and flow cytometry (**F**) assays were performed to evaluate the effects of circ-TRIO overexpression on the proliferative activities of TNBC cells. **G** Cell cycle-related proteins were examined after circ-TRIO overexpression. Transwell assays were performed to determine the influence of circ-TRIO overexpression on migration (**H**) and invasion (**I**) abilities. **J** Wound-healing assays were also performed to identify the migration ability. **K** Important proteins involved in the EMT pathway were examined after circ-TRIO knockdown. *p < 0.05; **p < 0.01; ***p < 0.001.

Since we proved that the knockdown of circ-TRIO inhibited the proliferation, migration, and invasion of TNBC cells in vitro, the effects of circ-TRIO overexpression were also evaluated. The circ-TRIO overexpression vector (circ-TRIO OV) and its control vector pLCDH were transfected into TNBC cells, and the efficiencies were evaluated by microscopy, qRT‒PCR and FISH assays (Fig. 3A, B). Moreover, the mRNA expression of the TRIO gene in TNBC cells was detected, and no significant changes were observed after circ-TRIO overexpression (Fig. S1B). As shown in Fig. 3C, D, the MTT and colony formation assays illustrated that the overexpression of circ-TRIO could significantly promote the proliferation rate of TNBC cells. Furthermore, the EDU and flow cytometry assays indicated that the proliferative activities of TNBC cells were enhanced (Fig. 3E, F). The proteins that participated in the G1/S phase of the cell cycle were also detected by a western blot analysis, which demonstrated that circ-TRIO could promote the proliferation of TNBC cells by regulating the cell cycle (Fig. 3G). Furthermore, the effects of circ-TRIO overexpression on TNBC cell migration and invasion were examined, and similar results were obtained in the Transwell and wound healing assays (Fig. 3H–J). Finally, EMT markers in TNBC cells were detected, and a positive correlation between circ-TRIO expression and EMT pathway activation was found (Fig. 3K).

### Circ-TRIO acts as a miRNA sponge for miR-432-5p

**Fig. 4:**
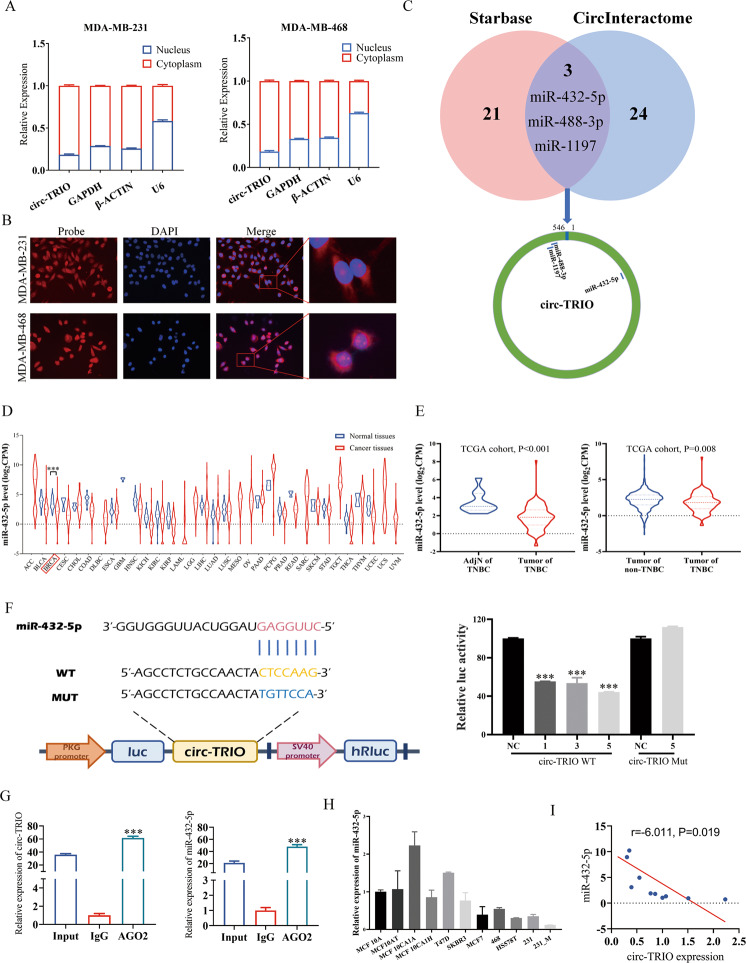
Circ-TRIO acts as a miRNA sponge of miR-432-5p. **A** Subcellular fractionation location assay of the circ-TRIO nuclear and cytoplasmic expression levels in TNBC cells. U6 was used as a nuclear marker, and GAPDH and β-actin were used as cytosolic markers. **B** Fluorescence in situ hybridization (FISH) with junction-specific probes was used to determine the localization of circ-TRIO in TNBC cells. **C** StarBase and CircInteractome databases were used to predict the potential binding miRNAs of circ-TRIO. **D** The expression of miR-432-5p in various cancer types based on TCGA database. **E** The expression of miR-432-5p among normal tissues adjacent to TNBC, TNBC tissues and non-TNBC tissues based on TCGA database. **F** The luciferase activity of circ-TRIO WT or circ-TRIO MUT after transfection with different concentrations of miR-432-5p mimics in 293 T cells. **G** Anti‐Ago2 RIP assay verified the combination potential between miR‐432-5p and circ-TRIO. **H** The expression of miR-432-5p in breast cancer cell lines. **I** The correlation between circ-TRIO expression and miR-432-5p expression. ***p < 0.001.

CircRNAs have been reported to function in cells via several different mechanisms, which are correlated with the intracellular locations of circRNAs, and the subcellular location of circ-TRIO in TNBC was detected by subcellular fractionation and FISH assays. As shown in Fig. 4A, B, we found that circ-TRIO was mainly located in the cytoplasm of TNBC cells, indicating that circ-TRIO has the potential to function as an endogenous competing RNA (ceRNA) and combine with miRNAs. To validate our hypothesis, two databases (Starbase [23] and CircInteractome [24]) were selected to filter potential circ-TRIO-targeted miRNAs. As shown in Fig. 4C, in total, 24 miRNAs from StarBase and 27 circRNAs from CircInteractome databases were identified, and only 3 miRNAs were both predicted by the databases, including miR-432-5p, miR-488-3p and miR-1197. The above filtered miRNAs were further analyzed based on TCGA [25] database to evaluate their potential functions, and the expression of miR-432-5p, miR-488-3p and miR-1197 is shown in Fig. 4D, E and Fig. S1C–F. We found that only miR-432-5p was downregulated in breast cancer tissues (Fig. 4D) and TNBC tissues (Fig. 4E), which was finally selected as a potential functional target of circ-TRIO.

To further determine whether circ-TRIO could directly combine with miR-432-5p, the wild-type (WT) and mutant (MUT) circ-TRIO full sequences were cloned into the pMIR-GLO vector. The combination site of miR-432-5p and the mutant sequence of circ-TRIO are shown in Fig. 4F. A luciferase assay revealed that miR-432-5p could significantly decrease the luciferase reporter activity of the wild type in a dose-dependent manner but not mutant circ-TRIO, suggesting that circ-TRIO could directly combine with miR-432-5p (Fig. 4F). In addition, the RIP assays proved that both circ-TRIO and miR-432-5p could be enriched by an anti-AGO2 antibody instead of anti-IgG (Fig. 4G), indicating that the association between circ-TRIO and miR-432-5p could participate in the RISC mechanism (RNA-induced silencing complex). Moreover, the expression of miR-432-5p in BC cells was further measured by qRT‒PCR and was decreased in breast cancer cells with higher malignancy (Fig. 4H). The association between circ-TRIO and miR-432-5p expression was also evaluated, and a significant negative correlation was found (Fig. 4I). Taken together, our results demonstrate that circ-TRIO might function as a ceRNA for miR-432-5p in TNBC cells.

### Overexpression of miR-432-5p inhibits breast cancer progression and reverses the oncogenic roles of circ-TRIO in TNBC

**Fig. 5:**
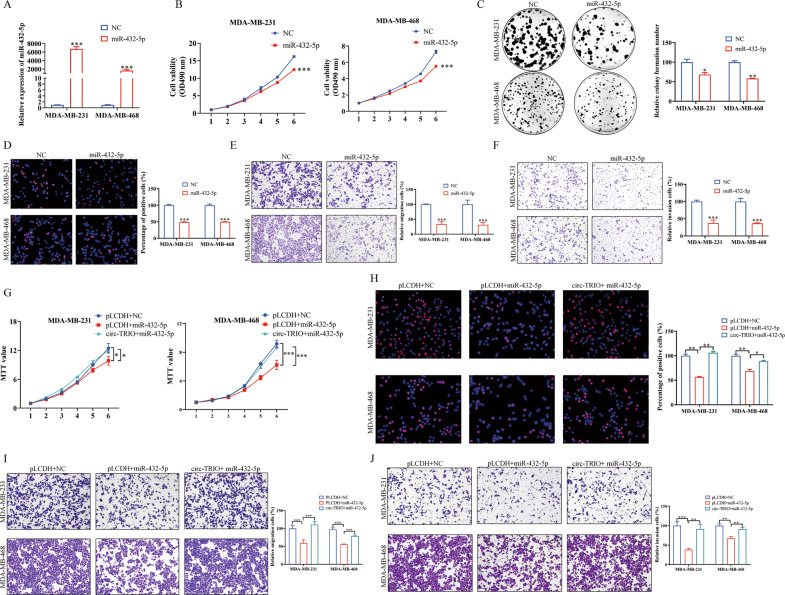
Overexpression of miR-432-5p inhibits breast cancer progression and reverses the oncogenic effects of circ-TRIO in TNBC. **A** The efficiency of miR-432-5p overexpression in TNBC cells. MTT (**B**), colony formation (**C**), and EdU (**D**) assays verified the effects of miR-432-5p overexpression on the TNBC proliferation rate. The migration (**E**) and invasion (**F**) abilities of MDA-MB-231 and MDA-MB-468 cell lines transfected with miR-432-5p mimics were evaluated using Transwell assays. MTT (**G**), EdU (**H**), and Transwell (**I, J**) assays revealed that the overexpression of circ-TRIO could reverse the effects of miR-432-5p. *p < 0.05; **p < 0.01; ***p < 0.001.

Since we proved that miR-432-5p could combine with circ-TRIO, we investigated whether miR-432-5p was a functional target of circ-TRIO. A qRT‒PCR assay was first used to evaluate the overexpression efficiency of miR-432-5p mimics (Fig. 5A). Using MTT, colony formation and EdU assays, we found that the overexpression of miR-432-5p inhibited the proliferation ability of TNBC cells (Fig. 5B–D). Moreover, the Transwell assays proved that a high expression of miR-432-5p significantly impaired the migration and invasion capabilities of TNBC cells (Fig. 5E, F). To further investigate whether circ-TRIO plays oncogenic roles by competing with miR-432-5p, both circ-TRIO and miR-432-5p were cotransfected into TNBC cells, and the rescue experiments showed that the overexpression of miR-432-5p could attenuate the functions of circ-TRIO in TNBC (Fig. 5G–J).

### CCDC58 is a direct target of miR-432-5p and is regulated by circ-TRIO

**Fig. 6:**
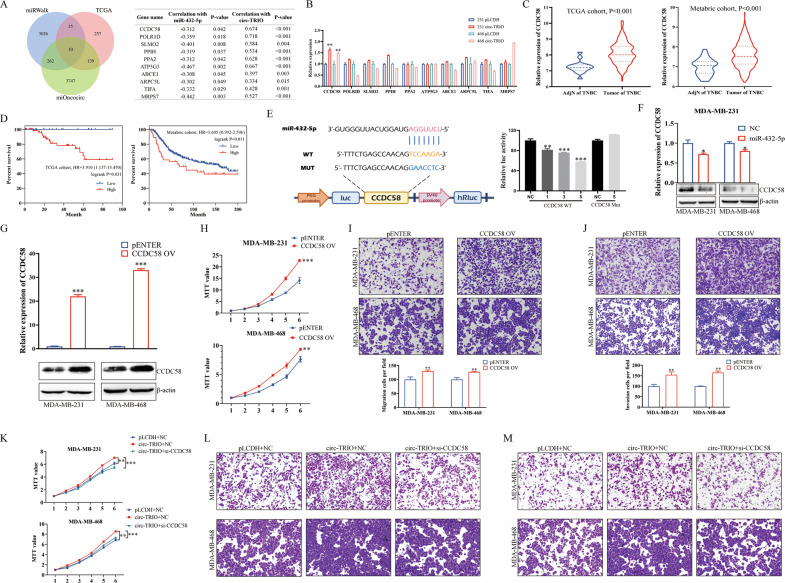
CCDC58 is a direct target of miR-432-5p and is regulated by circ-TRIO. **A** MiRWalk, TCGA and miOncocirc databases were used to screen potential functional target genes of miR-432-5p. The correlations between the filtered genes and miR-432-5p or circ-TRIO are also provided. **B** qRT‒PCR was performed to detect the expression of the filtered genes in circ-TRIO-overexpressing TNBC cells. **C** The expression of CCDC58 in normal tissues adjacent to TNBC and tumor tissues of TNBC was analyzed based on TCGA and Metabric database. **D** The correlations between CCDC58 expression and prognoses of TNBC patients were evaluated by TCGA and Metabric databases. **E** Luciferase activity of CCDC58 3ʹ UTR-WT or CCDC58 3ʹ UTR-MUT after transfection with different concentrations of miR-432-5p mimics in 293 T cells. **F** The mRNA and protein expression levels of CCDC58 after miR-432-5p overexpression were measured by qRT‒PCR and a western blot analysis. **G** The efficiency of CCDC58 overexpression was detected at the mRNA and protein levels. The effects of CCDC58 overexpression on proliferation (**H**), migration (**I**) and invasion (**J**) abilities were examined. MTT (**K**) and Transwell (**L**, **M**) assays revealed that the knockdown of CCDC58 could reverse the effects of circ-TRIO overexpression. *p < 0.05; **p < 0.01; ***p < 0.001.

It has been reported that the competing combination of circRNA and miRNA disturbs the expression of downstream genes. We further determined the functional targets of both circ-TRIO and miR-432-5p based on the miRWalk [26], TCGA [25] and miRCirc [27] databases. As shown in Fig. 6A, the miRWalk database was used to predict the potential target genes of miR-432-5p, and 3333 genes were filtered. Then, the TCGA database was analyzed, and 441 genes that were negatively correlated with the expression of miR-432-5p were selected. Moreover, 4,158 genes that were coexpressed with circ-TRIO were identified based on the miOncocirc database. Finally, 10 genes were ultimately filtered, and their correlations with miR-432-5p and circ-TRIO are shown in Fig. 6A, right. The relative expression of the 10 genes was further evaluated in TNBC cells with circ-TRIO expression, and CCDC58 was selected due to its increased expression in both MDA-MB-231 and MDA-MB-468 cells (Fig. 6B). The correlations between CCDC58 and miR-432-5p or circ-TRIO are shown in Fig. S1G. The relative expression and prognostic value of CCDC58 in TNBC were also evaluated by TCGA and Metabric [28] databases, showing that CCDC58 might be an oncogenic gene, which is consistent with our results (Fig. 6C, D).

To prove that CCDC58 is a direct target of miR-432-5p, dual-luciferase reporter gene assays were performed to explore the interaction between miR-432-5p and the 3ʹUTR of CCDC58. As shown in Fig. 6E, the full-length 3ʹUTR of CCDC58 with WT or MUT miR-432-5p binding sites was subcloned into pMIR-GLO vectors. Our results showed that miR-432-5p could decrease the luciferase activity in a dose-dependent manner in the WT group but not the MUT group. Furthermore, the effects of miR-432-5p overexpression on the CCDC58 mRNA and protein levels were examined (Fig. 6F). Taken together, our results demonstrate that miR-432-5p could inhibit CCDC58 by directly targeting the 3ʹUTR.

To explore the potential functions of CCDC58, the CCDC58 overexpression vector and its control vector pENTER were transfected into TNBC cells, and the efficiency was examined at both the mRNA and protein levels (Fig. 6G). Then, MTT and Transwell assays were performed and indicated that CCDC58 could upregulate the proliferation, migration and invasion abilities of TNBC cells (Fig. 6H–J). Moreover, since we proved that CCDC58 is the direct target of miR-432-5p, we further evaluated whether CCDC58 is also a functional target of circ-TRIO. As shown in Fig. S1H, the circ-TRIO overexpression vector and CCDC58 siRNA were transfected into TNBC cells, and CCDC58 expression was detected at the mRNA and protein levels. Further MTT and Transwell assays showed that the knockdown of CCDC58 could reverse the promoting effects of circ-TRIO overexpression in TNBC cells (Fig. 6K–M, Fig. S1I), proving that circ-TRIO could promote the progression of TNBC by regulating the downstream miR-432-5p/CCDC58 axis.

### Circ-TRIO facilitates the proliferation and metastasis of TNBC cells in vivo

**Fig. 7:**
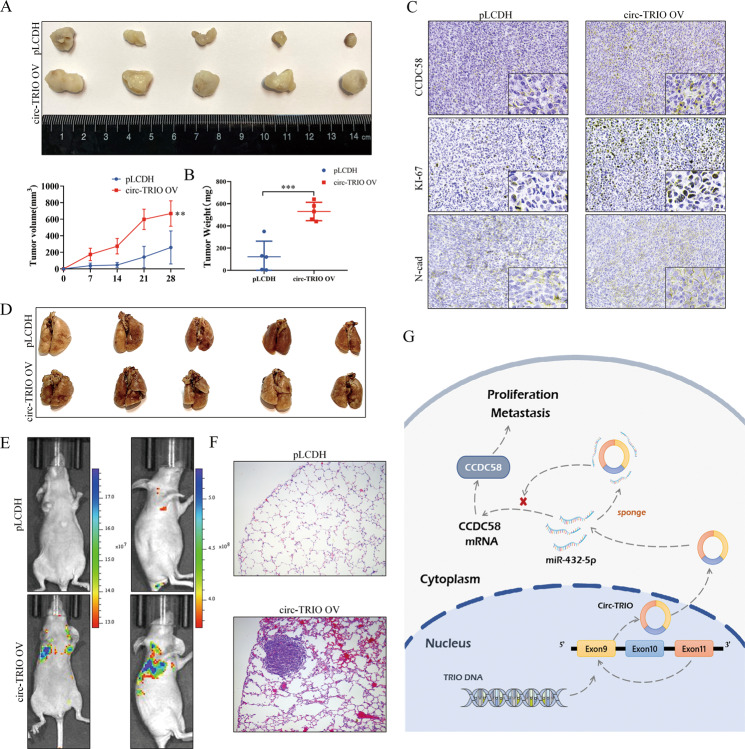
Circ-TRIO facilitates the proliferation and metastasis of TNBC cells in vivo. **A** Upper panel, images of xenograft tumors obtained from BALB/c nude mice at the endpoint. Lower panel, growth curve of the tumor volumes, which were measured every 7 days after the initial first week. **B** Weights of xenograft tumors at the endpoint. **C** CCDC58, Ki-67, and N-cad staining by IHC in xenograft tumors. **D** Images of lung metastatic nodules from BALB/c nude mice at the endpoint. **E** Animal in vivo imaging technology showing tumor metastasis. **F** H&E staining showed tumor metastasis. **G** Schematic diagram showing the mechanism by which circ-TRIO exerts its impacts on proliferation and metastasis in TNBC. **p < 0.01; ***p < 0.001.

To evaluate the tumor-promoting roles of circ-TRIO in vivo, MDA-MB-231 cells transfected with circ-TRIO overexpression and control vectors were first seeded subcutaneously into BALB/c nude mice to establish TNBC xenograft models. As shown in Fig. 7A, the tumors were measured 7, 14, 21 and 28 days after the tumor injection, and the tumor volume in the circ-TRIO group was significantly increased compared with that in the pLCDH group. Moreover, the tumor weight was increased in the circ-TRIO group (Fig. 7B). The tumor tissues in both groups were collected, and IHC assays of CCDC58, KI-67 and N-cad were performed. As shown in Fig. 7C, the expression levels of CCDC58, KI-67 and N-cad were remarkably upregulated in the circ-TRIO group, which is consistent with our in vitro assays. Furthermore, TNBC cells were injected into nude mice through the tail vein to establish a lung metastasis model. As shown in Fig. 7D and Fig. S1J, we found that the lung metastatic nodules in the circ-TRIO group were significantly increased, which was further validated by the in vivo fluorescence imaging (Fig. 7E and Fig. S1K) and HE staining (Fig. 7F) assays. Overall, these data indicate that increased circ-TRIO expression could efficiently promote the proliferation and metastasis of TNBC cells via the regulation of the miR-432-5p/CCDC58 axis (Fig. 7G).

## Discussion

With the rapid development of biological information and high-throughput sequencing, an increasing number of circRNAs have been found in recent years, and tumor-related circRNAs have become hotspots of research [29]. It is acknowledged that circRNAs can participate in tumorigenesis and progression via several mechanisms, including sponging microRNAs, interacting with RBPs, protein scaffolding and regulating gene transcription [30, 31]. Emerging studies have revealed that the mechanism of miRNA sponging widely exists in various types of tumors, such as ovarian cancer [32], prostate cancer [33], and gastric cancer [34]. In breast cancer, circRNAs have also been regarded as a class of noncoding RNAs related to tumorigenesis. For example, Sang et al. discovered that circRNA_0025202 could regulate tamoxifen sensitivity and tumor progression by regulating the miR-182-5p/FOXO3a axis in breast cancer [35]. Wang et al. suggested that circPGR could function as a ceRNA to promote the cell growth of estrogen receptor-positive breast cancer [17]. Wang et al. found that circACTN4 could interact with FUBP1 to promote the tumorigenesis and progression of breast cancer [36]. However, to date, the biological functions of the circRNAs associated with TNBC remain largely unknown.

To screen functional circRNAs correlated with the progression of TNBC, RNA-seq and public data were used, and a novel circRNA termed circ-TRIO was filtered; circ-TRIO is generated from exons 9-11 of the TRIO gene. TRIO, which is a triple functional domain protein, harbors 2 GEF domains and a protein serine kinase domain [37] and has been implicated in multiple processes of cancer in recent years. For instance, the knockdown of TRIO suppresses the migration and invasion of cervical cancer cells [38], and the activation of the NOTCH-DAB1-ABL-RHOGEF protein TRIO promotes the invasion and metastasis of colorectal cancer [39]. In this research, we found that circ-TRIO was highly expressed in tumor tissues and metastatic TNBC cells and increased in TNBC cells compared with hormone-positive BC cells, indicating that circ-TRIO might play vital roles in the malignant behaviors of TNBC. Studies have proven that circRNAs are equipped with highly conservative, highly stable, stage-specific tissue development and expression specificity in different diseases, which allows circRNAs to become not only potential biomarkers of prognosis but also molecular target genes for individualized treatment [40]. To explore the functions and mechanisms of circ-TRIO, we first verified the basic circular characteristics of circ-TRIO and demonstrated that circ-TRIO was more stable to withstand exonuclease-mediated degradation and had a longer half-life than its corresponding linear transcripts, proving that the circular form of circ-TRIO is endogenous in TNBC cells.

Based on previous studies, circRNAs can confer progression abilities to cancer cells and perform special regulatory functions in cancer proliferation, migration, and invasion processes [18, 41]; thus, functional experiments of circ-TRIO were also performed by in vitro and in vivo assays. We demonstrated that the knockdown of circ-TRIO could inhibit the proliferation, migration and invasion of TNBC cells, while the overexpression of circ-TRIO had the opposite effects, revealing the oncogenic roles of circ-TRIO in TNBC. Furthermore, by qPCR analyses of the expression of circ-TRIO in TNBC patients, we found that high circ-TRIO expression led to a poor prognosis, including poor overall survival and disease-free survival, indicating that circ-TRIO plays a vital role in patients suffering from TNBC. To further explore the molecular mechanism of circ-TRIO, we first identified the subcellular location of circ-TRIO because it has been reported that the mechanisms of circRNAs are correlated with their intracellular location [33, 42, 43]. Based on the results of the subcellular fractionation and FISH assays, circ-TRIO was located in the cytoplasm of TNBC cells, indicating that circ-TRIO might have the potential to sponge miRNAs. To date, numerous studies have shown that acting as miRNA sponges is among the most significant approaches by which circRNAs exert their functions [44–46]. CiRS-7 was the first circRNA that has been proven to exert its functions by acting as a miRNA sponge, possessing more than 70 conventional binding sites for miR-7 [47]. Subsequently, a series of studies indicated that ciRS-7 could act as a negative regulator of miR-7 and upregulate the expression of its target genes [48, 49]. To verify whether circ-TRIO could act as a miRNA sponge, StarBase, CircInteractome and TCGA were used to screen the potential target miRNAs of circ-TRIO, and miR-432-5p was finally identified. Subsequently, a dual-luciferase reporter gene assay was used to identify the interaction between circ-TRIO and miR-432-5p, demonstrating that circ-TRIO could sponge miR-432-5p.

As a type of noncoding RNA, miRNAs participate in a variety of cellular biological processes and can combine with the 3ʹUTR and regulate the expression of target genes [50, 51]. It is acknowledged that the controlled expression of miRNAs is indispensable for normal cells, while the aberrant expression of miRNAs may lead to diseases, including cancers [52, 53]. As reported, miR-432-5p could be regarded as a tumor inhibitor in human cancers; for example, DRAIC could promote the growth of breast cancer by sponging miR-432-5p to upregulate SLBP [54], circ-ZNF609 could target miR-432-5p to regulate the expression of LRRC1 to promote cholangiocarcinoma [55], and LINC01783 could facilitate cell proliferation, migration and invasion in non-small cell lung cancer by targeting miR-432-5p [56]. However, the underlying function of miR-432-5p in TNBC has not been reported. In our study, we found that miR-432-5p could inhibit the malignant behaviors of TNBC cells. Furthermore, rescue experiments were performed and verified that the upregulation of circ-TRIO could reverse the function of miR-432-5p, indicating that circ-TRIO could act as a sponge and inhibit the function of miR-432-5p, which, in turn, upregulates the expression of downstream mRNAs.

CCDC58 (coiled-coil domain containing 58), also known as MIX23 (mitochondrial matrix import Factor 23), is widely expressed in a variety of tissues in mice and humans, including skeletal muscle, the heart, and the brain [57, 58]. The physiological functions of CCDC58 have been discovered in recent high-throughput interaction studies; CCDC58 is localized to mitochondria and has been found to interact with numerous IMS proteins, including ATPase family gene 3 like 2 (AFG3L2), apoptosis inducing factor mitochondria associated 1 (AIFM1), SCO1 synthesis, and cytochrome c [59, 60]. CCDC58 was also identified as one of the few cellular proteins whose absence provides resistance against the intracellular bacterium Ehrlichia chaffeensis, the cause of monocytic ehrlichiosis [61]. Increased CCDC58 levels have been identified as an unfavorable prognostic factor in endometrial hyperplasia and liver and urothelial cancer [62], suggesting that CCDC58 might be an oncogenic gene, but the functions of CCDC58 in cancers remain elusive. In our study, we screened miR-432-5p as a target gene by the miRWalk, TCGA and miRCocirc databases, and their direct combination was verified by a dual-luciferase reporter assay. Further cell functional experiments showed that CCDC58 could promote the proliferation, migration and invasion abilities of TNBC cells, and the knockdown of CCDC58 could reverse the functions of circ-TRIO, proving that CCDC58 is a functional oncogenic gene in TNBC cells.

In conclusion, our study illustrates that circ-TRIO functions as an oncogenic circRNA to facilitate the proliferation and metastasis of TNBC cells by regulating the miR-432-5p/CCDC58 axis, and the expression of circ-TRIO is correlated with the recurrence and prognoses of TNBC patients. The newly identified circ-TRIO broadens our insight into the underlying mechanisms of TNBC and represents a potential prognostic and therapeutic target for the treatment of patients with TNBC.

## Supplementary information

Supplementary Figure legend

Supplementary Figure S.1

Supplementary Table S.1

Supplementary Table S.2

Supplementary Table S.3

Original western blots

Agreement from all authors for adding authors

Reproducibility checklist

## Data Availability

The data that supports the findings of this study are available from the corresponding author upon reasonable request.
