# Supplementary material for: Circ-TRIO promotes TNBC progression by regulating the miR-432-5p/CCDC58 axis
Source: Cell Death Dis. 2022 Sep 8;13(9):776. doi: 10.1038/s41419-022-05216-7 (PMC9458743; doi:10.1038/s41419-022-05216-7)
Supplement: Supplementary file 1 — Supplementary Figure legend [file 41419_2022_5216_MOESM1_ESM.docx]

**Figure S.1. A.** Effects of circ-TRIO knockdown on TRIO mRNA expression. **B.** Effects of circ-TRIO overexpression on TRIO mRNA expression. **C.** The expression of miR-488-3p in various cancer types based on TCGA database. **D.** The expression of miR-488-3p among normal tissues adjacent to TNBC, TNBC tissues and non-TNBC tissues based on TCGA database. **E.** The expression of miR-1197 in various cancer types based on TCGA database. **F.** The expression of miR-1197 among normal tissues adjacent to TNBC, TNBC tissues and non-TNBC tissues based on TCGA database. **G.** The correlations between CCDC58 expression and miR-432-5p or circ-TRIO expression. **H.** Circ-TRIO was overexpressed, CCDC58 was knocked down, and the expression of CCDC58 was detected at the mRNA and protein levels. **I.** Statistical charts of Figure 6.L and Figure 6.M. **J.** Quantitation of lung metastasis nodules. **K.** In vivo bioluminescent images at Day 0 were presented as the normalizing control. ns, nonsignificant; *p<0.05; **p<0.01; ***p < 0.001.
