# Supplementary figures and images for: Circ-TRIO promotes TNBC progression by regulating the miR-432-5p/CCDC58 axis
Source: Cell Death Dis. 2022 Sep 8;13(9):776. doi: 10.1038/s41419-022-05216-7 (PMC9458743; doi:10.1038/s41419-022-05216-7)

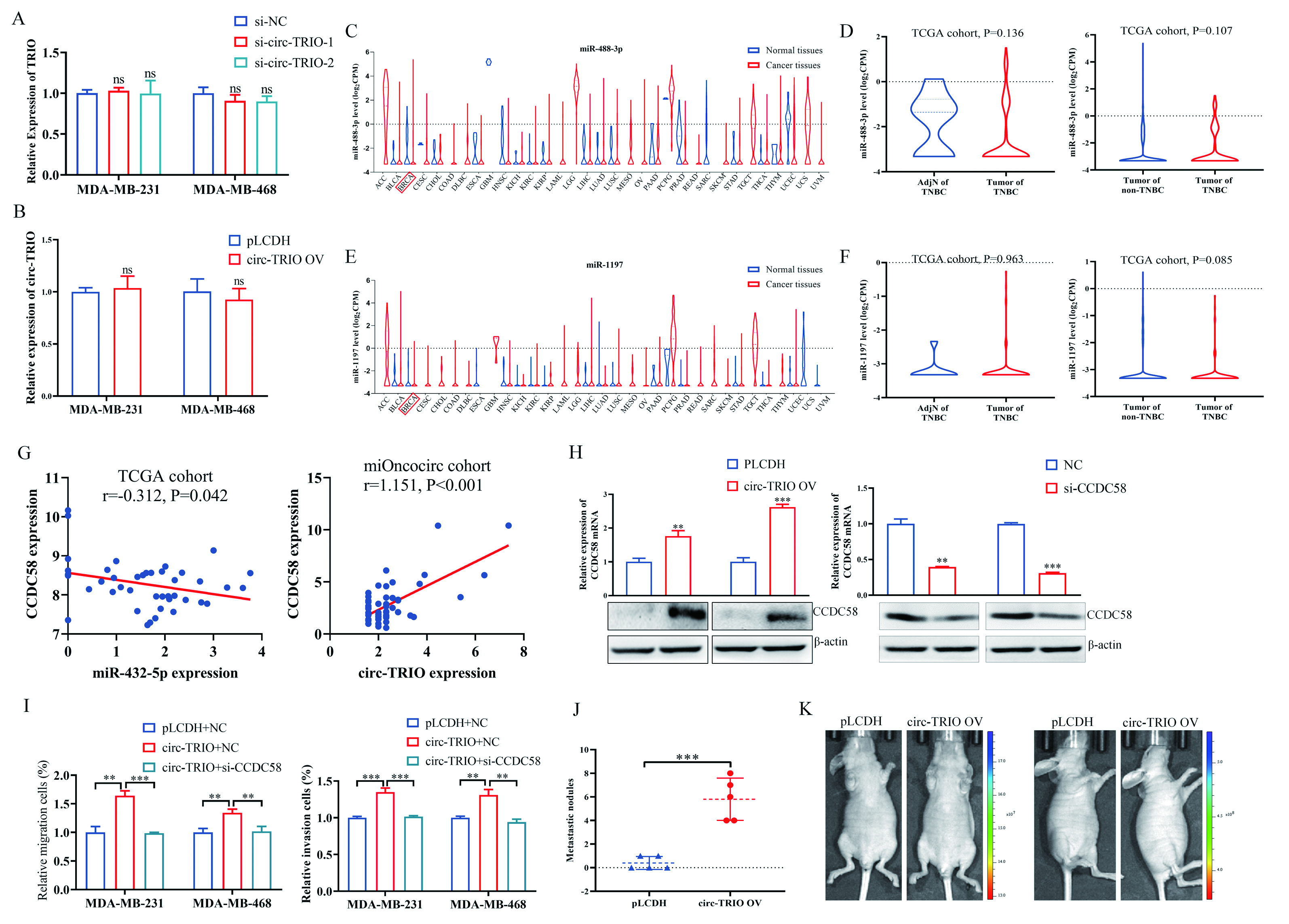

Supplement: Supplementary file 2 — Supplementary Figure S.1 [file 41419_2022_5216_MOESM2_ESM.jpg]
