# Supplementary material for: Circ-TRIO promotes TNBC progression by regulating the miR-432-5p/CCDC58 axis
Source: Cell Death Dis. 2022 Sep 8;13(9):776. doi: 10.1038/s41419-022-05216-7 (PMC9458743; doi:10.1038/s41419-022-05216-7)
Supplement: Supplementary file 6 — Original western blots [file 41419_2022_5216_MOESM6_ESM.pptx]

## Slide 1
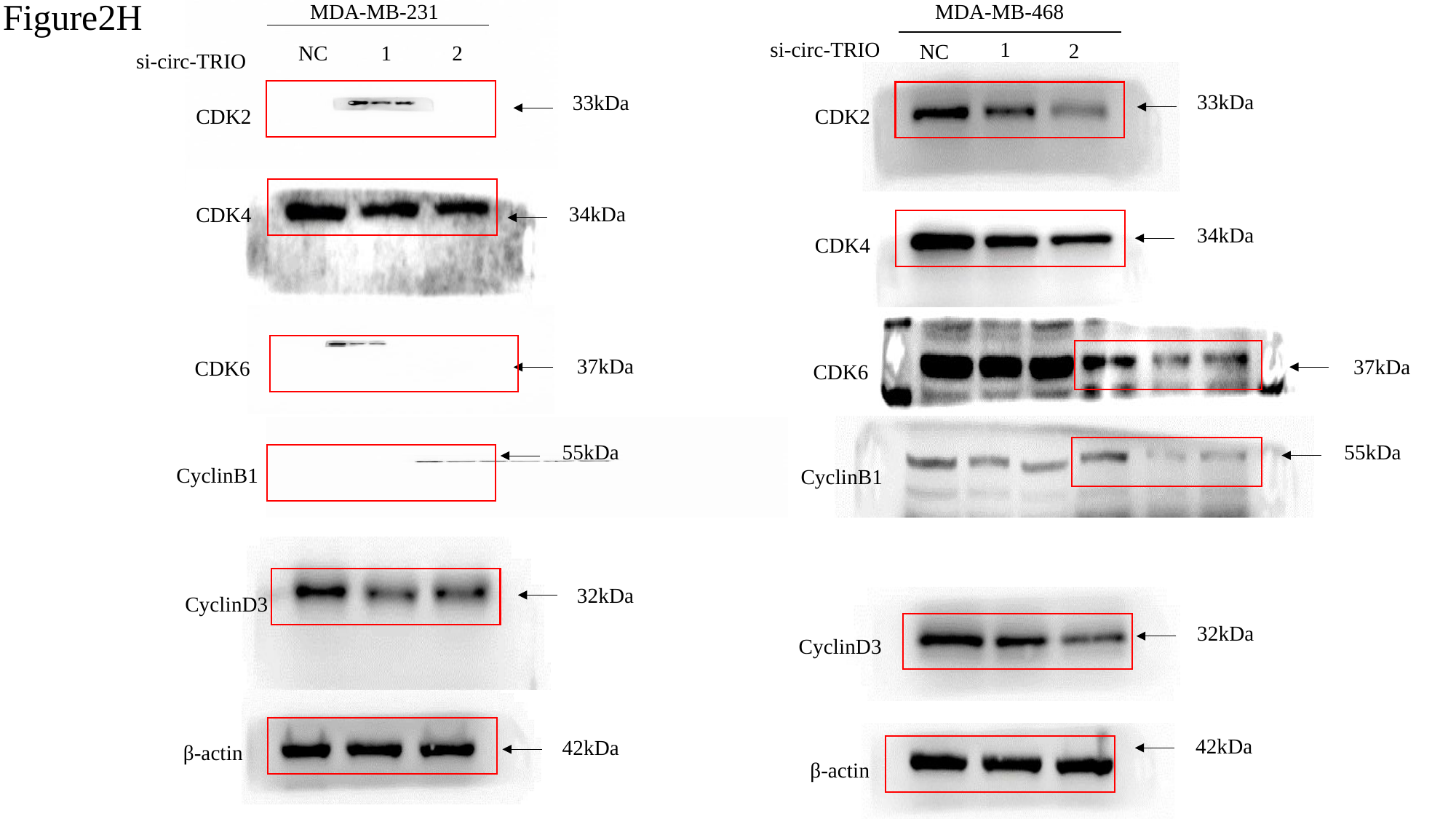

Figure2H
MDA-MB-231
MDA-MB-468
1
si-circ-TRIO
2
NC
1
2
NC
si-circ-TRIO
33kDa
33kDa
CDK2
CDK2
34kDa
CDK4
34kDa
CDK4
37kDa
37kDa
CDK6
CDK6
55kDa
55kDa
CyclinB1
CyclinB1
32kDa
CyclinD3
32kDa
CyclinD3
42kDa
42kDa
β-actin
β-actin

## Slide 2
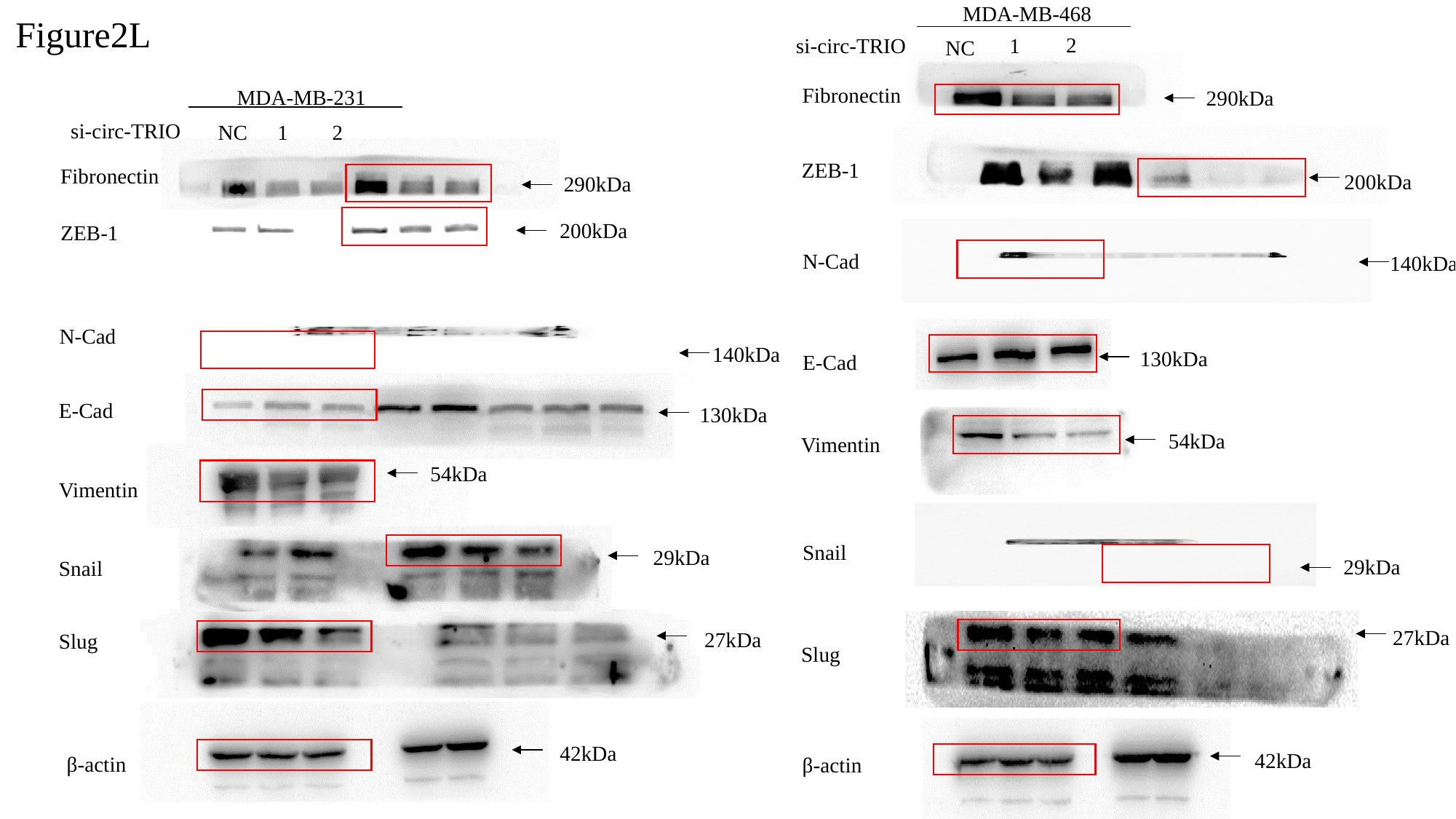

MDA-MB-468
Figure2L
2
si-circ-TRIO
1
NC
Fibronectin
MDA-MB-231
290kDa
si-circ-TRIO
NC
1
2
ZEB-1
Fibronectin
200kDa
290kDa
200kDa
ZEB-1
N-Cad
140kDa
N-Cad
140kDa
130kDa
E-Cad
E-Cad
130kDa
54kDa
Vimentin
54kDa
Vimentin
Snail
29kDa
29kDa
Snail
27kDa
27kDa
Slug
Slug
42kDa
42kDa
β-actin
β-actin

## Slide 3
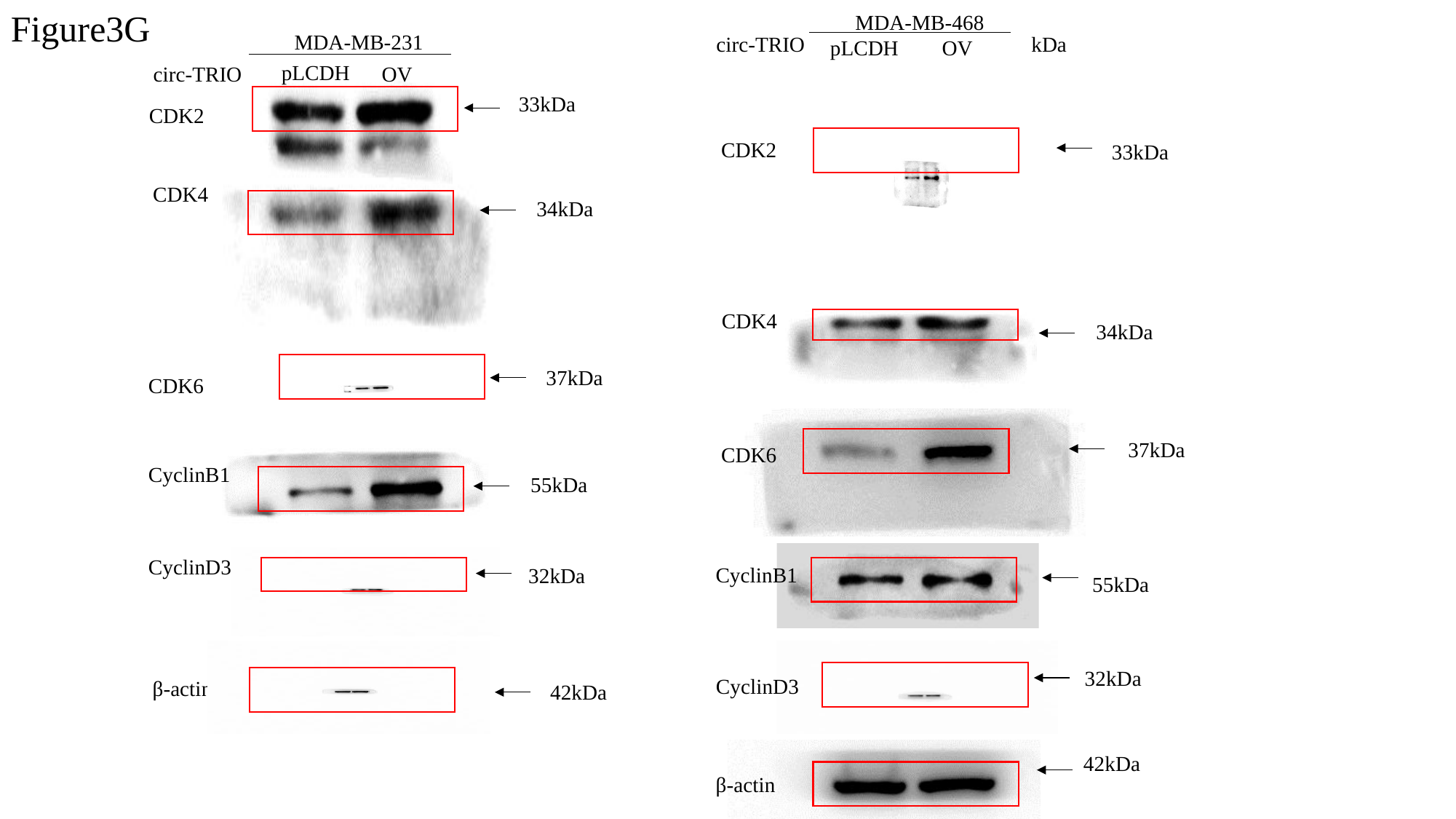

Figure3G
MDA-MB-468
MDA-MB-231
circ-TRIO
kDa
pLCDH
OV
pLCDH
circ-TRIO
OV
33kDa
CDK2
CDK2
33kDa
CDK4
34kDa
CDK4
34kDa
37kDa
CDK6
37kDa
CDK6
CyclinB1
55kDa
CyclinD3
CyclinB1
32kDa
55kDa
32kDa
CyclinD3
β-actin
42kDa
42kDa
β-actin

## Slide 4
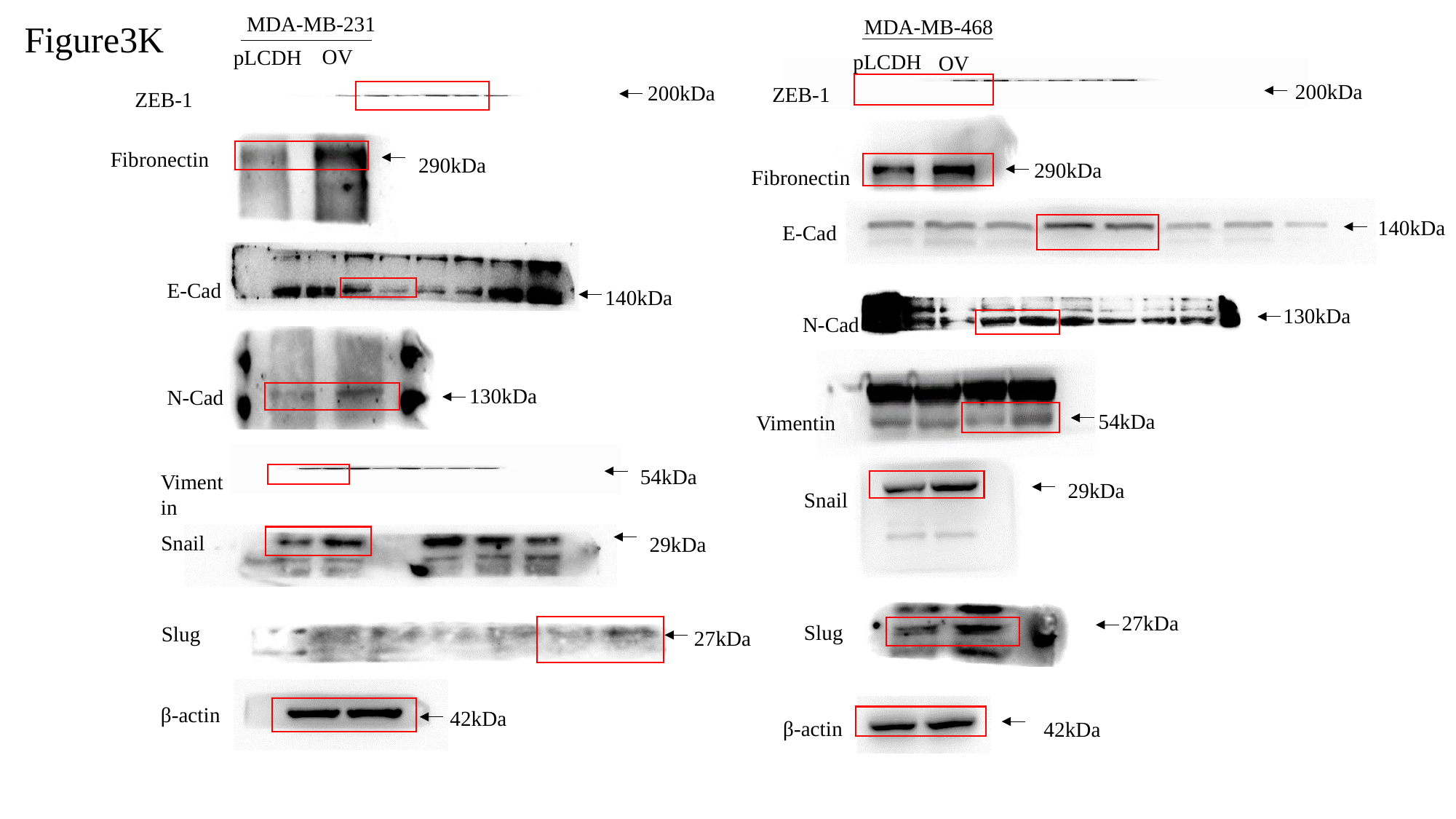

MDA-MB-231
MDA-MB-468
Figure3K
OV
pLCDH
pLCDH
OV
200kDa
200kDa
ZEB-1
ZEB-1
Fibronectin
290kDa
290kDa
Fibronectin
140kDa
E-Cad
E-Cad
140kDa
130kDa
N-Cad
130kDa
N-Cad
54kDa
Vimentin
54kDa
Vimentin
29kDa
Snail
Snail
29kDa
27kDa
Slug
Slug
27kDa
β-actin
42kDa
β-actin
42kDa

## Slide 5
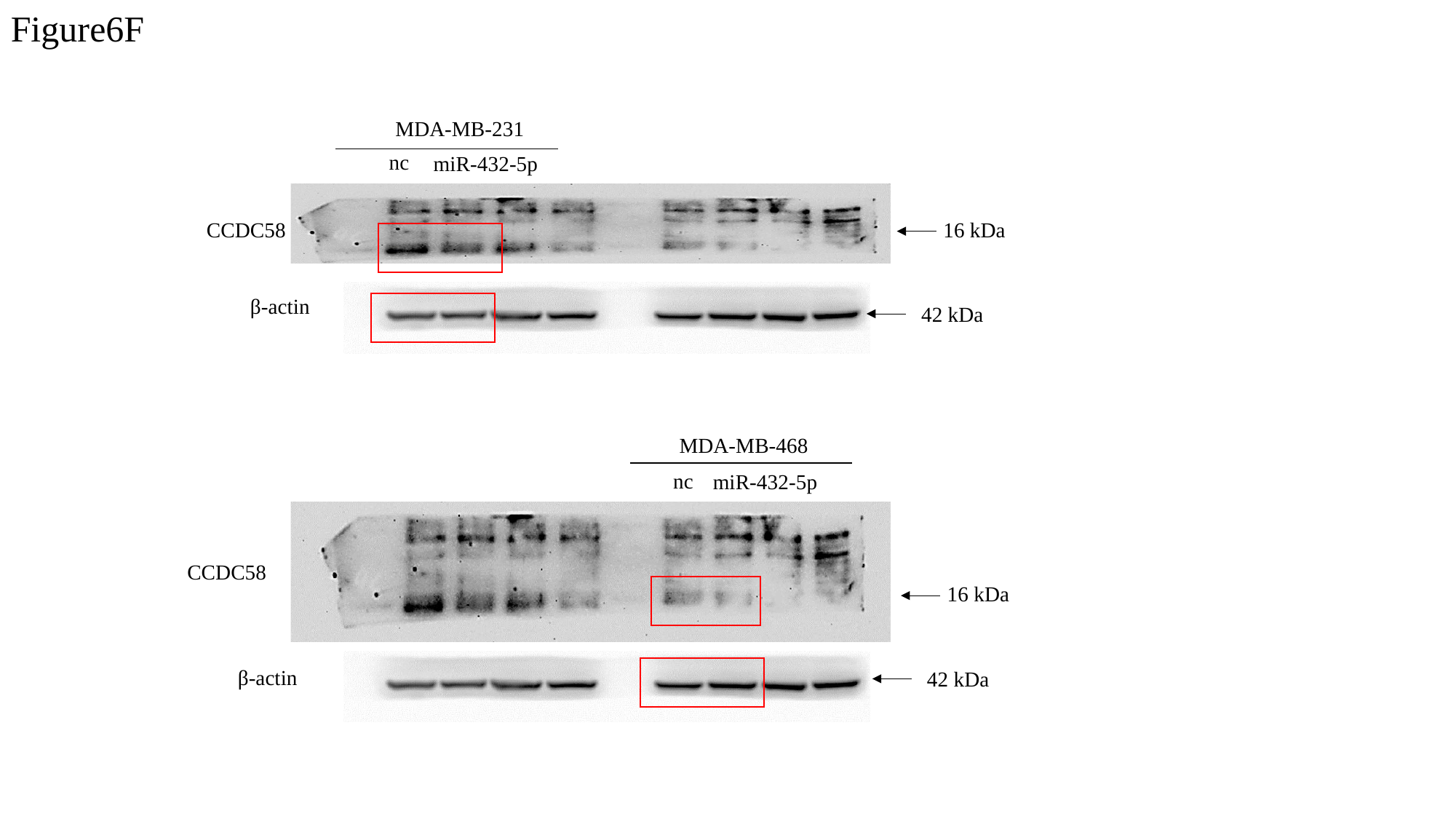

Figure6F
F
MDA-MB-231
nc
miR-432-5p
CCDC58
16 kDa
β-actin
42 kDa
MDA-MB-468
nc
miR-432-5p
CCDC58
16 kDa
β-actin
42 kDa

## Slide 6
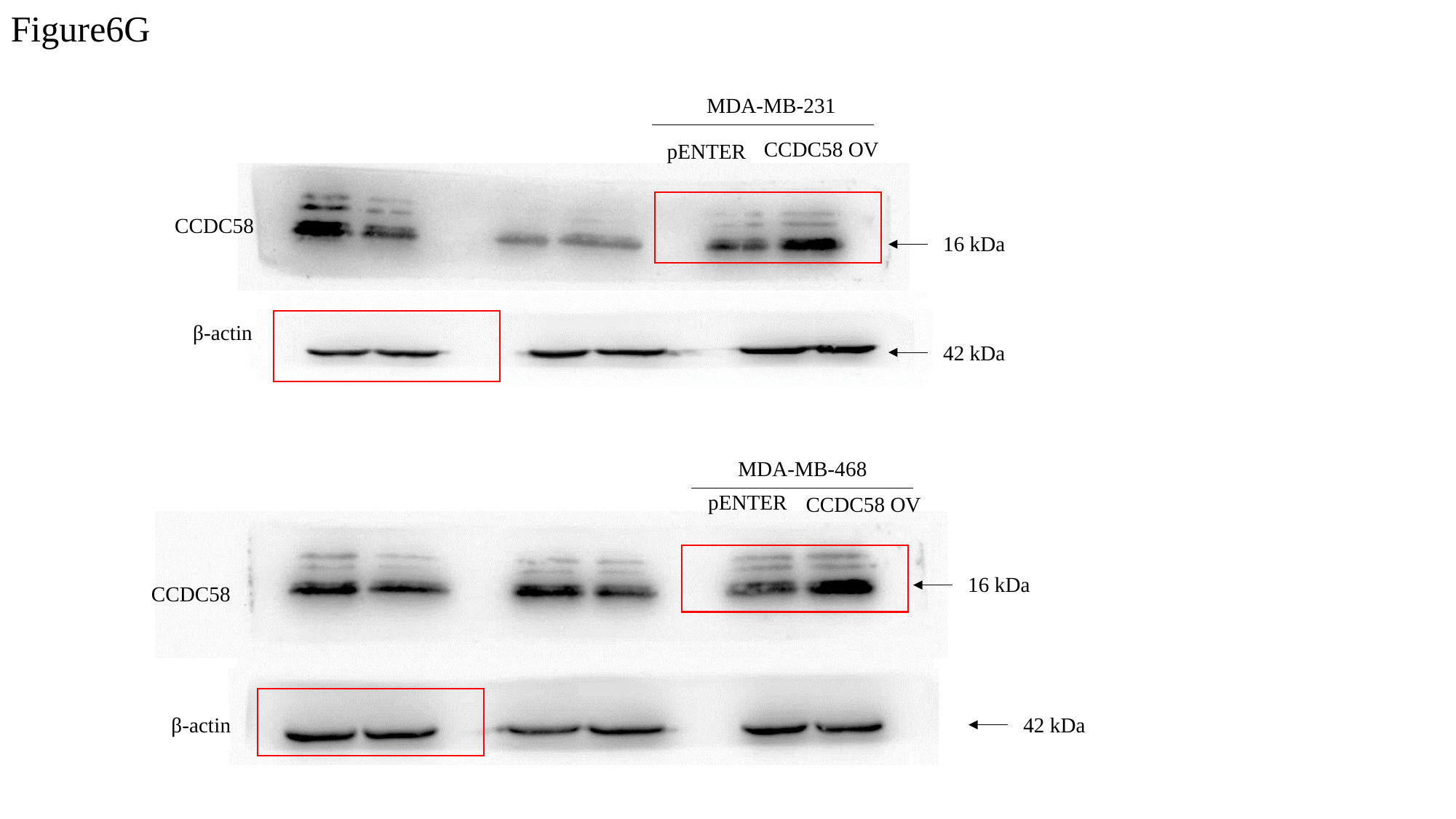

Figure6G
MDA-MB-231
CCDC58 OV
pENTER
CCDC58
16 kDa
β-actin
42 kDa
MDA-MB-468
pENTER
CCDC58 OV
16 kDa
CCDC58
β-actin
42 kDa

## Slide 7
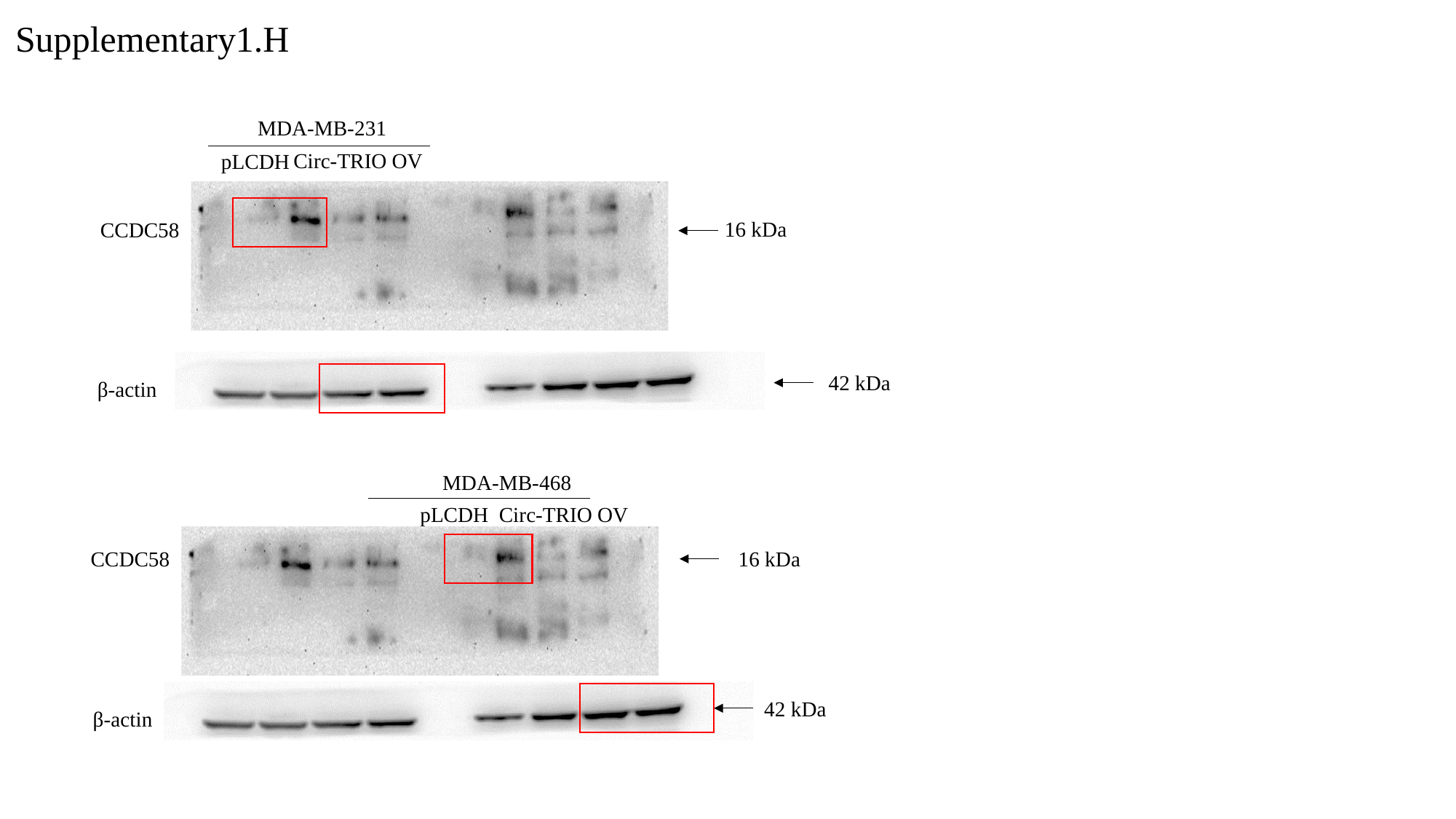

Supplementary1.H
MDA-MB-231
Circ-TRIO OV
pLCDH
16 kDa
CCDC58
42 kDa
β-actin
MDA-MB-468
pLCDH
Circ-TRIO OV
CCDC58
16 kDa
42 kDa
β-actin

## Slide 8
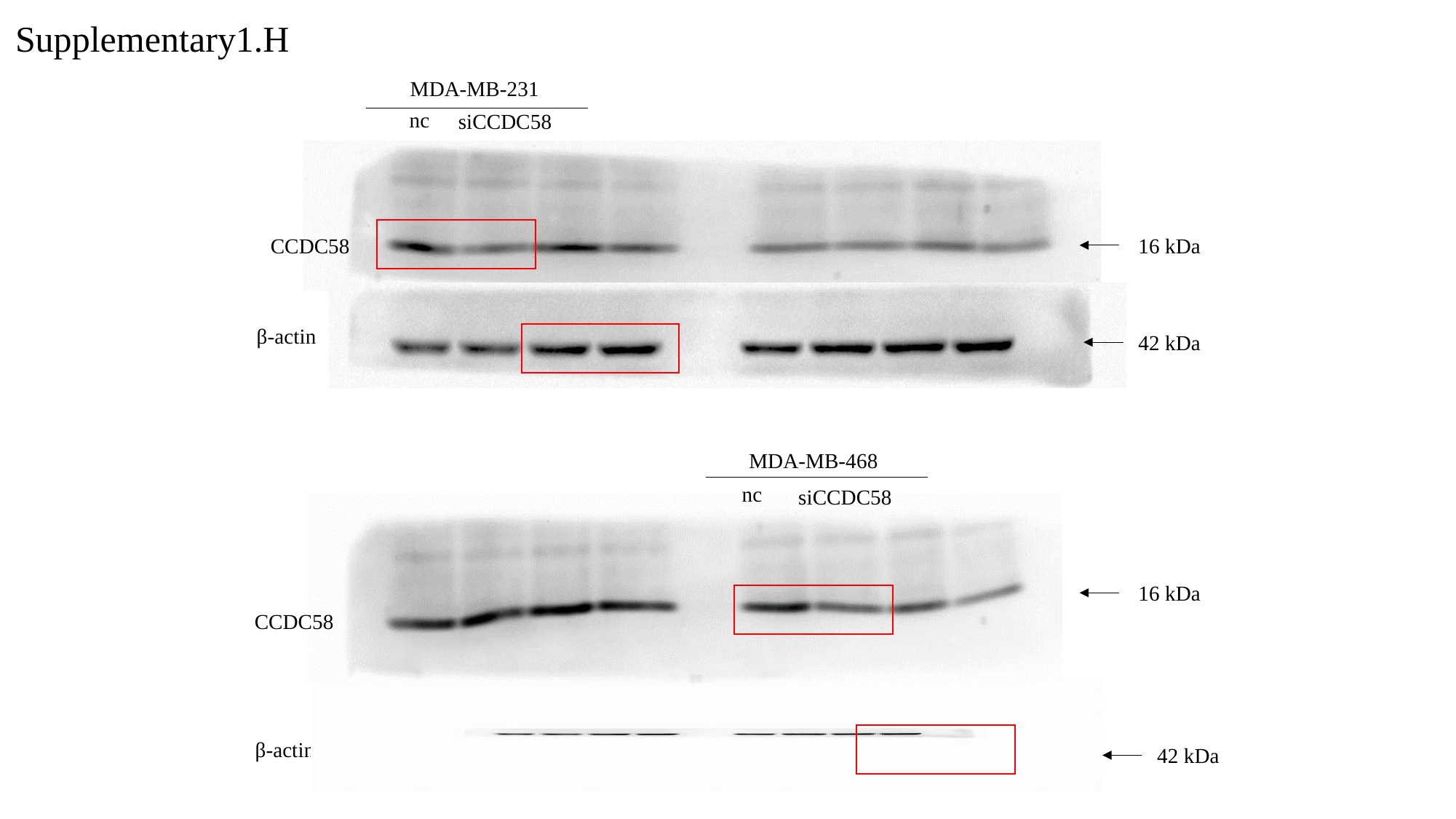

Supplementary1.H
MDA-MB-231
nc
siCCDC58
CCDC58
16 kDa
β-actin
42 kDa
MDA-MB-468
nc
siCCDC58
16 kDa
CCDC58
β-actin
42 kDa
