# Supplementary material for: Circ-TRIO promotes TNBC progression by regulating the miR-432-5p/CCDC58 axis
Source: Cell Death Dis. 2022 Sep 8;13(9):776. doi: 10.1038/s41419-022-05216-7 (PMC9458743; doi:10.1038/s41419-022-05216-7)
Supplement: Supplementary file 7 — Agreement from all authors for adding authors [file 41419_2022_5216_MOESM7_ESM.pdf]

Response from Zekun Wang

Re: Adding Xiaoli Kong as another co-author in the manuscirpt titled 'Circ-TRIO promotes TNBC progression by regulating the miR-432-5p/CCDC58 axis'.

发件人：王泽坤 <zekun69@163.com>
时　间：2022年8月23日 (星期二) 下午4：46
收件人：qifengy_sdu <qifengy_sdu@163.com>

纯文本 |

邮件可翻译为中文 立即翻译

Thank you for your letter.
I agree with the addition of Xiaoli Kong as a co-author in this manuscript considering her substantial contribution.
Zekun Wang

---- Replied Message ----

Dear Zekun Wang,

During the revison period of the manuscirpt titled 'Circ-TRIO promotes TNBC progression by regulating the miR-432-5p/CCDC58 axis', manuscript number 'CDDIS-22-1431RR', Xiaoli Kong contributed much in the revised version of this manuscript. After we received the reviewer's comments, she was responsible for designing and arranging experiments needed to solve the questions raised by reviewers. Hence, I'm writing the email to request agreement from you for adding Xiaoli Kong as another co-author in this manuscript.

Looking for your reply!

Your sincerely,

Qifeng Yang

Response from Yaming Li

Re: Adding Xiaoli Kong as another co-author in the manuscirpt titled 'Circ-TRIO promotes TNBC progression by regulating the miR-432-5p/CCDC58 axis'.

发件人：doctorliyaming <doctorliyaming@gmail.com>
时　间：2022年8月24日 (星期三) 上午8：24
收件人：qifengy_sdu <qifengy_sdu@163.com>

纯文本 |

邮件可翻译为中文 立即翻译

Thank you for your letter.
I agree with the addition of Xiaoli Kong as a co-author in this manuscript considering her substantial contribution.
Yaming Li

doctorliyaming
doctorliyaming@gmail.com

Dear Yaming Li,

During the revison period of the manuscirpt titled 'Circ-TRIO promotes TNBC progression by regulating the miR-432-5p/CCDC58 axis', manuscript number 'CDDIS-22-1431RR', Xiaoli Kong contributed much in the revised version of this manuscript. After we received the reviewer's comments, she was responsible for designing and arranging experiments needed to solve the questions raised by reviewers. Hence, I'm writing the email to request agreement from you for adding Xiaoli Kong as another co-author in this manuscript.

Looking for your reply!

Your sincerely,

Qifeng Yang

Response from Jingwen Yang

回复：Adding Xiaoli Kong as another co-author in the manuscirpt titled 'Circ-TRIO promotes TNBC progression by regulating the miR-432-5p/CCDC58 axis'.

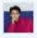

发件人：杨婧雯 <1844600426@qq.com>
时　间：2022年8月23日 (星期二) 下午5：17
收件人：qifengy_sdu <qifengy_sdu@163.com>

这不是腾讯公司的官方邮件。 请勿轻信密保、汇款、中奖信息，勿轻易拨打陌生电话。 举报垃圾邮件 网站安全云检测

邮件可翻译为中文 立即翻译

Thank you for your letter.
I agree with the addition of Xiaoli Kong as a co-author in this manuscript considering her substantial contribution.
Jingwen Yang

------------ 原始邮件 ------------

Dear Jingwen Yang,

During the revison period of the manuscirpt titled 'Circ-TRIO promotes TNBC progression by regulating the miR-432-5p/CCDC58 axis', manuscript number 'CDDIS-22-1431RR', Xiaoli Kong contributed much in the revised version of this manuscript. After we received the reviewer's comments, she was responsible for designing and arranging experiments needed to solve the questions raised by reviewers. Hence, I'm writing the email to request agreement from you for adding Xiaoli Kong as another co-author in this manuscript.

Looking for your reply!

Your sincerely,

Qifeng Yang

Response from Yiran Liang

回复: Adding Xiaoli Kong as another co-author in the manuscirpt titled 'Circ-TRIO promotes TNBC progression by regulating the miR-432-5p/CCDC58 axis'.

发件人: liangyiran321 <liangyiran321@sina.com>
时　间: 2022年8月23日 (星期二) 下午4 : 43
收件人: qifengy_sdu <qifengy_sdu@163.com>

纯文本 |

邮件可翻译为中文 立即翻译

Thank you for your letter.
I agree with the addition of Xiaoli Kong as a co-author in this manuscript considering her substantial contribution.
Yiran Liang

--
发自新浪邮箱客户端

Dear Yiran Liang,

During the revison period of the manuscirpt titled 'Circ-TRIO promotes TNBC progression by regulating the miR-432-5p/CCDC58 axis', manuscript number 'CDDIS-22-1431RR', Xiaoli Kong contributed much in the revised version of this manuscript. After we received the reviewer's comments, she was responsible for designing and arranging experiments needed to solve the questions raised by reviewers. Hence, I'm writing the email to request agreement from you for adding Xiaoli Kong as another co-author in this manuscript.

Looking for your reply!

Your sincerely,

Qifeng Yang

Response from Xiaolong Wang

Re:Adding Xiaoli Kong as another co-author in the manuscirpt titled 'Circ-TRIO promotes TNBC progression by regulating the miR-432-5p/CCDC58 axis'.

发件人: wxlxs12366 <wxlxs12366@126.com>
时　间: 2022年8月23日 (星期二) 下午4 : 57
收件人: qifengy_sdu <qifengy_sdu@163.com>

纯文本 |

邮件可翻译为中文 立即翻译

Thank you for your letter.
I agree with the addition of Xiaoli Kong as a co-author in this manuscript considering her substantial contribution.
Xiaolong Wang

---- Replied Message ----

Dear Xiaolong Wang,

During the revison period of the manuscirpt titled 'Circ-TRIO promotes TNBC progression by regulating the miR-432-5p/CCDC58 axis', manuscript number 'CDDIS-22-1431RR', Xiaoli Kong contributed much in the revised version of this manuscript. After we received the reviewer's comments, she was responsible for designing and arranging experiments needed to solve the questions raised by reviewers. Hence, I'm writing the email to request agreement from you for adding Xiaoli Kong as another co-author in this manuscript.

Looking for your reply!

Your sincerely,

Qifeng Yang

Response from Ning Zhang

Re:Adding Xiaoli Kong as another co-author in the manuscirpt titled 'Circ-TRIO promotes TNBC progression by regulating the miR-432-5p/CCDC58 axis'.

发件人: zhangning0816 <zhangning0816@163.com>
时　间: 2022年8月23日 (星期二) 下午5 : 12
收件人: qifengy_sdu <qifengy_sdu@163.com>

纯文本 |

邮件可翻译为中文 立即翻译

Thank you for your letter.
I agree with the addition of Xiaoli Kong as a co-author in this manuscript considering her substantial contribution.

Best,
Ning Zhang

--
Ning Zhang, M.D.
Department of Breast Surgery,
Qilu Hospital of Shandong University,
Jinan, China, 250012

Replied Message

Dear Ning Zhang,

During the revison period of the manuscirpt titled 'Circ-TRIO promotes TNBC progression by regulating the miR-432-5p/CCDC58 axis', manuscript number 'CDDIS-22-1431RR', Xiaoli Kong contributed much in the revised version of this manuscript. After we received the reviewer's comments, she was responsible for designing and arranging experiments needed to solve the questions raised by reviewers. Hence, I'm writing the email to request agreement from you for adding Xiaoli Kong as another co-author in this manuscript.

Looking for your reply!

Your sincerely,

Qifeng Yang

Response from Bing Chen

Re:Adding Xiaoli Kong as another co-author in the manuscirpt titled 'Circ-TRIO promotes TNBC progression by regulating the miR-432-5p/CCDC58 axis'.

发件人: 18560088179 <18560088179@163.com>
时　间: 2022年8月23日 (星期二) 下午5 : 04
收件人: qifengy_sdu <qifengy_sdu@163.com>

纯文本 |

邮件可翻译为中文 立即翻译

Thank you for your letter.
I agree with the addition of Xiaoli Kong as a co-author in this manuscript considering her substantial contribution.
Bing Chen

---- Replied Message ----

Dear Bing Chen,

During the revison period of the manuscirpt titled 'Circ-TRIO promotes TNBC progression by regulating the miR-432-5p/CCDC58 axis', manuscript number 'CDDIS-22-1431RR', Xiaoli Kong contributed much in the revised version of this manuscript. After we received the reviewer's comments, she was responsible for designing and arranging experiments needed to solve the questions raised by reviewers. Hence, I'm writing the email to request agreement from you for adding Xiaoli Kong as another co-author in this manuscript.

Looking for your reply!

Your sincerely,

Qifeng Yang

Response from Lijuan Wang

Re:Adding Kongxiao Li as another co-author in the manuscirpt titled 'Circ-TRIO promotes TNBC progression by regulating the miR-432-5p/CCDC58 axis'.

发件人: doctorwanglijuan <doctorwanglijuan@163.com>
时　间: 2022年8月23日 (星期二) 下午5 : 01
收件人: qifengy_sdu <qifengy_sdu@163.com>

纯文本 |

邮件可翻译为中文 立即翻译

Thank you for your letter.
I agree with the addition of Xiaoli Kong as a co-author in this manuscript considering her substantial contribution.
Lijuan Wang

---- Replied Message ----

Dear Lijuan Wang,

During the revison period of the manuscirpt titled 'Circ-TRIO promotes TNBC progression by regulating the miR-432-5p/CCDC58 axis', manuscript number 'CDDIS-22-1431RR', Kongxiao Li contributed much in the revised version of this manuscript. After we received the reviewer's comments, she was responsible for designing and arranging experiments needed to solve the questions raised by reviewers. Hence, I'm writing the email to request agreement from you for adding Kongxiao Li as another co-author in this manuscript.

Looking for your reply!

Your sincerely,

Qifeng Yang

Response from Wenjing Zhao

Re:Adding Kongxiao Li as another co-author in the manuscirpt titled 'Circ-TRIO promotes TNBC progression by regulating the miR-432-5p/CCDC58 axis'.

发件人: 赵文静 <zhaowenjing0911@163.com>
时　间: 2022年8月23日 (星期二) 下午5 : 01
收件人: qifengy_sdu <qifengy_sdu@163.com>

纯文本 |

邮件可翻译为中文 立即翻译

Thank you for your letter.
I agree with the addition of Xiaoli Kong as a co-author in this manuscript considering her substantial contribution.

Wenjing Zhao

---- Replied Message ----

Dear Wenjing Zhao,

During the revison period of the manuscirpt titled 'Circ-TRIO promotes TNBC progression by regulating the miR-432-5p/CCDC58 axis', manuscript number 'CDDIS-22-1431RR', Kongxiao Li contributed much in the revised version of this manuscript. After we received the reviewer's comments, she was responsible for designing and arranging experiments needed to solve the questions raised by reviewers. Hence, I'm writing the email to request agreement from you for adding Kongxiao Li as another co-author in this manuscript.

Looking for your reply!

Your sincerely,

Qifeng Yang

Response from Xiaoli Kong

Re: Adding Xiaoli Kong as another co-author in the manuscirpt titled 'Circ-TRIO promotes TNBC progression by regulating the miR-432-5p/CCDC58 axis'.

发件人: 15953129882 <15953129882@163.com>
时　间: 2022年8月23日 (星期二) 下午7 : 10
收件人: qifengy_sdu <qifengy_sdu@163.com>

纯文本 |

邮件可翻译为中文 立即翻译

Thanks for your approval，I'd like to be a co-author.
Xiaoli Kong

---- Replied Message ----

Dear Xiaoli Kong,

I'm the corresponding author of manuscript titled 'Circ-TRIO promotes TNBC progression by regulating the miR-432-5p/CCDC58 axis', manuscript number 'CDDIS-22-1431RR', I would like to add you as one of the co-authors of this manuscript based on your contribution in the revised version of this manuscript. Hence, I'm writing the email to request agreement for adding you as a co-author in this manuscript.

Looking for your reply!

Your sincerely,

Qifeng Yang
